# Molecular basis for blue light-dependent phosphorylation of *Arabidopsis* cryptochrome 2

**DOI:** 10.1038/ncomms15234

**Published:** 2017-05-11

**Authors:** Qing Liu, Qin Wang, Weixian Deng, Xu Wang, Mingxin Piao, Dawei Cai, Yaxing Li, William D. Barshop, Xiaolan Yu, Tingting Zhou, Bin Liu, Yoshito Oka, James Wohlschlegel, Zecheng Zuo, Chentao Lin

**Affiliations:** 1 Basic Forestry and Proteomics Research Center, Fujian Agriculture and Forestry University, Fuzhou 350002, China; 2 College of Plant Science, Jilin University, Changchun 130062, China; 3 Department of Molecular, Cell & Developmental Biology, University of California, Los Angeles, California 90095, USA; 4 Department of Biological Chemistry, University of California, Los Angeles, California 90095, USA; 5 Institute of Crop Sciences, Chinese Academy of Agricultural Sciences, Beijing 10081, China

## Abstract

Plant cryptochromes undergo blue light-dependent phosphorylation to regulate their activity and abundance, but the protein kinases that phosphorylate plant cryptochromes have remained unclear. Here we show that photoexcited *Arabidopsis* cryptochrome 2 (CRY2) is phosphorylated *in vivo* on as many as 24 different residues, including 7 major phosphoserines. We demonstrate that four closely related Photoregulatory Protein Kinases (previously referred to as MUT9-like kinases) interact with and phosphorylate photoexcited CRY2. Analyses of the *ppk123* and *ppk124* triple mutants and *amiR*^*4k*^ artificial microRNA-expressing lines demonstrate that PPKs catalyse blue light-dependent CRY2 phosphorylation to both activate and destabilize the photoreceptor. Phenotypic analyses of these mutant lines indicate that PPKs may have additional substrates, including those involved in the phytochrome signal transduction pathway. These results reveal a mechanism underlying the co-action of cryptochromes and phytochromes to coordinate plant growth and development in response to different wavelengths of solar radiation in nature.

## Abstract (web summary)

Plant cryptochromes are regulated by blue-light dependent phosphorylation. Here the authors map the *in vivo* phosphorylation sites of *Arabidopsis* cryptochrome 2 and identify four closely related kinases that act to both activate and destabilize the receptor in response to blue light.

Cryptochromes are photolyase-like flavoproteins that act as blue-light receptors or core components of the circadian clock in animals and plants^1^,^2^. Cryptochromes have two recognizable domains, a photolyase homologous region (PHR) domain and a cryptochrome C-terminal extension (CCE) domain. The *Arabidopsis* genome encodes two cryptochromes, CRY1 and CRY2, that regulate primarily photomorphogenic development and photoperiodic flowering, respectively^3^,^4^,^5^. Plant cryptochromes undergo photoexcitation to become physiologically active homodimers^6^,^7^,^8^, which interact with CRY-signalling partners such as cryptochrome-interacting bHLHs (CIBs) and suppressor of PhyA 1 (SPA1) to control blue light-responsive gene expression changes and plant developmental responses^9^,^10^,^11^,^12^,^13^,^14^. We have previously shown that *Arabidopsis* CRY1 and CRY2 both undergo blue light-dependent phosphorylation^8^,^15^,^16^,^17^,^18^. The blue light-dependent CRY2 phosphorylation occurs primarily in the nucleus^17^,^19^ and enhances CRY2 activity, polyubiquitination, and proteolysis, by altering conformation of the photoreceptor^15^,^18^,^20^,^21^. We have recently shown that the negative regulators of cryptochromes, blue-light inhibitors of cryptochromes 1 and 2 (BIC1 and BIC2) suppress dimerization, phosphorylation, degradation and physiological activities of *Arabidopsis* CRY2 (ref. 8).

Cryptochrome phosphorylation has been extensively studied in the mammalian model system. For example, at least five different protein kinases have been reported to be involved in the light-independent phosphorylation of at least nine serine residues of mouse cryptochromes in response to timing and metabolic cues, including adenosine monophosphate-activated protein kinase (AMPK), glycogen synthase kinase 3β (GSK3β), dual-specificity tyrosine-phosphorylated and regulated kinase 1A (DYRK1A), DNA-dependent protein kinase (DNA-PK), mitogen-activated protein kinase (MAPK) and casein kinase I ɛ (CKIɛ)^22^,^23^,^24^,^25^,^26^. Inhibition of cryptochrome phosphorylation results in increased stability and altered activities of cryptochromes, leading to distorted period lengths or phases of the circadian clock of mammalian cells. Significantly less is known about light-dependent phosphorylation of plant cryptochromes, despite the fact that protein phosphorylation was first discovered in *Arabidopsis* CRY2 (ref. 16). It has been reported that *Arabidopsis* casein kinase 1.3 and 1.4 (CK1.3 and CK1.4) catalyse blue light-enhanced phosphorylation of residues S587 and T603 of CRY2 (ref. 27), and that adenosine monophosphate-activated protein kinase (AMPK) may also play a role in the phosphorylation and stability of *Arabidopsis* CRY1 in response to nitrogen availability^28^. However, it has remained unclear what residues of CRY2 are phosphorylated *in vivo* and whether these two protein kinases are responsible for the phosphorylation of those residues of cryptochromes in plants. We have recently reported that at least three serine residues (S598, S599, S605) in the CCE domain of *Arabidopsis* CRY2 undergo blue light-dependent phosphorylation and that mutations in those residues reduced the physiological activity but increased protein stability of the mutant CRY2 in plants^18^. Despite this progress, it has remained elusive how many residues of CRY2, in addition to S598, S599 and S605, undergo blue light-dependent phosphorylation *in vivo* and what protein kinases catalyse phosphorylation of those residues.

Here, we map the *in vivo* phosphorylation sites of *Arabidopsis* cryptochrome 2 and identify four closely related kinases that catalyse blue light-specific phosphorylation of CRY2 to both activate and destabilize the photoreceptor in response to blue light.

## Results

### The *in vivo* phosphosites of *Arabidopsis* CRY2

**Figure 1:**
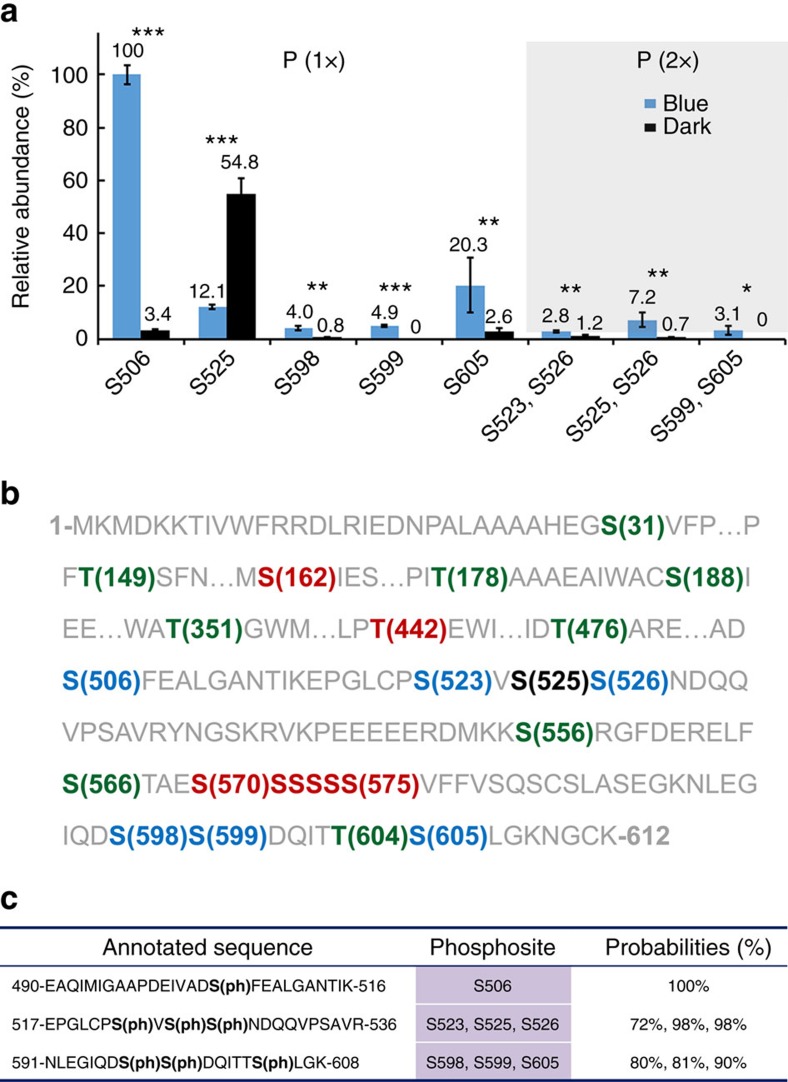
MS analyses of the blue light-dependent *in vivo* phosphosites of CRY2. (**a**) The relative abundance of major phosphopeptides detected in GFP-CRY2 purified from etiolated (Dark) seedlings exposed to blue light for 30 min (Blue: 30 μmol m^−2^ s^−1^) that are detected by at least three algorithms in at least three independent MS experiments. The intensity of the indicated phosphopeptide containing the respective phosphosite was determined by the label-free MS1 peak area of each phosphopeptide performed with Maxquant. Data represent three biological replicates, each contains both the light-treated samples (Blue) and the dark control (Black). The LFQ intensity of phosphopeptide derived from MaxQuant was normalized against the LFQ intensity of the GFP-CRY2 input, and converted to relative abundance (numbers shown on the top of each bar) by dividing with the mean (*n*=3) of the most abundant phosphopeptide (S506) detected, which was set to 100%. The s.d. are shown (*n*=3). The phosphopeptide exhibited significantly different abundance between dark and light conditions was indicated as *** (Student's *t*-test, *P*<0.001), ** (Student's *t*-test, *P*<0.01), or * (Student's *t*-test, *P*<0.05) on the top of bars. (**b**) Major and minor phosphosites of CRY2 (residues 1–612, with sequences containing no phosphosite omitted as indicated by …) detected in this study. The phosphosites of CRY2 detected by at least three experiments and recognizable by 3, 2 or 1 algorithms are denoted by blue, red or green colour, respectively. The blue light-repressed phosphosite (S525) is coloured by black. (**c**) Examples of phosphopeptides of CRY2 phosphorylated on the mono-phosphosite and tri-phosphosites. The maximum probability shown for each phosphosite is calculated by Andromeda algorithm integrated in MaxQuant.

Endogenous CRY2 is a low abundance protein making it difficult to purify for biochemical studies. Therefore, we purified the GFP-CRY2 fusion protein from overexpressing transgenic plants to enable the study of *in vivo* CRY2 phosphorylation, since GFP–CRY2 has the similar photobiochemical and photophysiological properties as the endogenous CRY2 (refs 18, 20, 21). We performed multiple independent experiments. In each experiment, we analysed two GFP-CRY2 samples, one collected from etiolated seedlings and another prepared from etiolated seedlings exposed to blue light (30 μmol m^−2^ s^−1^) for 30 min. We purified GFP–CRY2 using the GFP-trap-immunoprecipitation (IP) method, and analysed phosphorylated residues of CRY2 using the label-free quantitative mass spectrometry (MS) method. We detected phosphorylation on at least 18 serine residues and 6 threonine residues of CRY2 across the multiple experiments (Fig. 1b). Among those phosphorylated residues (phosphosites), seven phosphoserines (S506, S523, S525, S526, S598, S599, S605), including three previously reported phosphoserines (S598, S599, S605)^18^, were reproducibly detected at a statistically significant level (Student's *t*-test, *P*<0.01) by multiple phospholocalization algorithms in at least three independent experiments and were considered ‘major' phosphosites of CRY2 (Fig. 1b, Supplementary Table 1, Supplementary Figs 1 and 2). Five of those seven major phosphosites (S506, S525, S598, S599, S605) were detected as either single or doubly phosphorylated peptides, whereas two of them (S523, S526) were detected exclusively as the doubly and triply phosphorylated peptides distinguishable by the different retention times (Fig. 1a,c, Supplementary Table 1, Supplementary Figs 1 and 2). Consistent with the blue light dependency of CRY2 phosphorylation reported previously^8^,^16^,^17^,^18^, the relative abundance of most phosphosites of CRY2, except S525, increased significantly (Student's *t*-test, *P*<0.01) in response to blue light (Fig. 1a; Supplementary Table 1a). In addition to the seven major phosphosites (S506, S523, S525, S526, S598, S599, S605), the GFP–CRY2 protein purified from plants was also phosphorylated at other ‘minor' phosphosites (Fig. 1b), which we define as not being reproducibly identified across all experiments and having ambiguous phosphopeptide localization when analysed using multiple phospholocalization algorithms. These minor phosphosites may also represent *bona fide in vivo* phosphosites of CRY2 that contribute to the overall level of phosphorylation of CRY2. For example, phosphopeptides containing phosphorylated residues in the serine cluster (S570–S575) in the CCE domain of CRY2 were detected in GFP–CRY2 purified from light-grown plants (Fig. 1b), but did not meet all of the criteria necessary to classify them as major CRY2 phosphosites (Fig. 1a,b). However, the CRY2^6SA^ mutant, in which all six serine residues of the serine cluster were converted to alanines, exhibited markedly decreased phosphorylation and physiological activity in plants, indicating that either phosphorylation or structure of the serine cluster is important for CRY2 phosphorylation and function in plants^18^. The finding that CRY2 contains at least 24 phosphosites would be consistent with our previous hypothesis that the blue light-dependent phosphorylation of CRY2 may be needed to provide sufficient negative charges to electrostatically repel the CCE domain from the PHR domain to form an ‘open' conformation in response to light^15^,^18^,^20^,^21^.

### MUT9-like protein kinases associate with photoexcited CRY2

**Figure 2:**
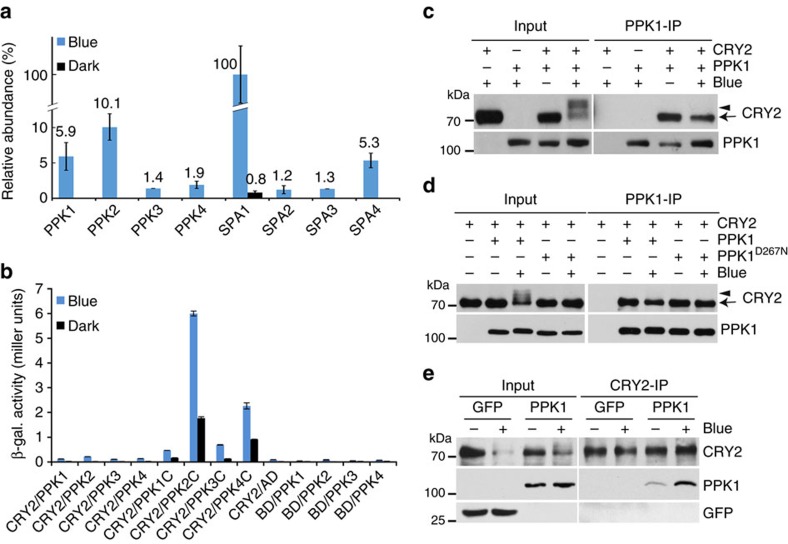
*In vivo* and *in vitro* assays showing the blue light-dependent interaction of CRY2 and PPKs. (**a**) CRY2-associated proteins detected by co-immunoprecipitation (co-IP) and subsequent mass spectrometric analysis. Two-week-old LD (long-day)-grown seedlings expressing *35S::GFP-CRY2* were transferred to darkness for one day, then exposed to blue light or kept in the dark for 60 min before sample collection. Data represent three biological replicates, each contains both the light-treated samples (Blue) and the dark control (Black). The LFQ intensity of indicated CRY2-interacting protein was calculated by the MaxLFQ algorithm integrated in the MaxQuant software, normalized against the LFQ intensity of immunoprecipitation bait (CRY2), and converted to relative abundance by the mean of the most abundant protein (SPA1) of the three biological replicates. The s.d. are shown (*n*=3). The lack of a bar/number in many ‘Dark' samples indicates that the indicated protein was not detected. (**b**) Results of yeast two-hybrid assays. The β-galactosidase activity of yeast cells expressing the CRY2 bait and PPK or PPKC (PPK C terminus) prey. Cells were grown in the dark, then exposed to blue light (30 μmol m^−2^ s^−1^) for 60 min. Binding domain and activation domain vector plasmids were used as negative controls. The s.d. are shown (*n*=3). (**c**–**e**) The CRY2–PPK1 interaction analysed by the Co-IP assays. (**c**) HEK293T cells co-expressing *CMV::Myc-CRY2* and *CMV::Flag-GFP–PPK1* were kept in the dark (Blue −) or exposed to blue light (30 μmol m^−2^ s^−1^, Blue +) for 2 h. Flag-GFP-PPK1 was immunoprecipitated by the anti-Flag antibody (PPK1-IP). The IP signal (PPK1) and the co-IP signal (CRY2) were analysed by the immunoblot, probed by anti-CRY2 (CRY2) or anti-GFP (PPK1), respectively. (**d**) Same as (c), except that *CMV::Myc-CRY2* was co-transfected with *CMV::Flag-GFP-PPK1*^*D267N*^. (**e**) Co-IP assays showing the blue light-dependent interaction between PPK1 and CRY2 in plants. 10-day-old etiolated seedlings expressing *ACT2::GFP-PPK1* were exposed to blue light (30 μmol m^−2^ s^−1^) for 20 min or kept in the dark. Plant extracts were immunoprecipitated by the anti-CRY2 antibody (CRY2-IP), and analysed by immunoblot probed with the anti-CRY2 (CRY2) or anti-GFP (PPK1) antibody, respectively. Transgenic plants expressing GFP was included as the negative control. In this and other figures alike, arrows or arrowheads indicate unphosphorylated or phosphorylated CRY2, respectively, unless otherwise indicated. Actin or a non-specific band (NS) recognized by anti-Flag antibody was used as the loading control.

MS analyses of the GFP-CRY2 recombinant protein purified from plants treated with different light conditions allowed not only detection of phosphosites of CRY2 but also the identification of proteins that co-purified with photoexcited GFP–CRY2. In addition to the known blue light-dependent CRY2-interacting proteins such as SPA1 (ref. 10), these experiments identified four closely related kinases that co-localize with CRY2 in the nucleus (Fig. 2a; Supplementary Fig. 3). Three of the four CRY2-associated kinases are previously named as MUT9-like kinases (MLK) and shown to redundantly regulate osmotic stress, circadian rhythm and photomorphogenic responses, although their kinase activity and substrate specificity have not been reported^29^,^30^. Given that these four CRY2-associated kinases constitute an independent clade evolutionarily related to but phylogenetically distinct from casein kinase 1 (CK1)^27^,^31^,^32^(Supplementary Fig. 4), that the name MLK has been previously used for a family of mammalian ‘Mixed-Lineage Kinases'^33^ unrelated to the MUT9-like kinases, that the substrates of MUT9-like kinases were previously unclear in plants^29^,^30^, and that the substrates of these four kinases have been identified by this and the accompany report as the key regulators of plant light responses (see later), we collectively refer to the MUT9-like kinases as photoregulatory protein kinases (PPK) and designated the corresponding genes as *PPK1* (AT3G13670), *PPK2* (AT5G18190), *PPK3* (AT3G03940) and *PPK4* (AT2G25760). PPKs and CK1s share ∼40% sequence identity in the kinase domain but diverge considerably in their C-terminal domains. Because the C-terminal domains of PPKs are highly conserved within the PPK clade, but exhibit little homology to the C-terminal domain of CK1 (Supplementary Fig. 5), they are referred to as the PPKC domain. PPKs also diverge from CK1s in their N-terminal extension which is found in none of the CK1 isomers (Supplementary Fig. 5).

All PPKs were detected only in the GFP–CRY2 complex isolated from plants exposed to blue light, but not in the GFP–CRY2 complex isolated from plants kept in the dark (Fig. 2a). PPKs were co-purified with GFP–CRY2 at levels lower than that of SPA1. For example, the relative amount of PPKs detected ranges from about 1.4 to 10.1% of the SPA1 detected in the same GFP–CRY2 complex isolated from light-treated plants (Fig. 2a). We further examined the CRY2–PPK interaction, using three different assays. In the first experiment, we tested the CRY2–PPK interaction using a yeast-two hybrid assay. Figure 2b shows that the PPKC domains of all PPKs exhibited clear blue light-dependent interaction with CRY2, although the full-length PPK proteins showed markedly lower affinity for CRY2. This result indicates that the highly conserved non-catalytic PPKC domains are the CRY2-interacting domains of PPKs. The different affinities of the full-length PPKs and the PPKC domain may also suggest that phosphorylation of CRY2 decreases its affinity for PPKs. Alternatively, different steric impacts of the full-length PPKs and PPKC domains or poor expression of the full-length PPKs in yeast cells may also explain this result. We next examined the CRY2–PPK1 interaction in human embryo kidney 293T (HEK293T) cells co-expressing epitope-tagged Flag-GFP-PPK1 and Myc-CRY2 using a co-immunoprecipitation (co-IP) assay. As we reported previously, the blue light-dependent phosphorylation of CRY2 resulted in an electrophoretic mobility shift and retarded migration of CRY2 in SDS–polyacrylamide gel electrophoresis (SDS–PAGE), as well as reduced relative abundance of the faster-migrating unphosphorylated CRY2 band^16^,^18^(Fig. 2c, Input). PPK1 co-precipitated with the fast-migrating unphosphorylated CRY2, but not the slow-migrating phosphorylated CRY2 from the light-treated cells (Fig. 2c, IP lanes). Although, we cannot completely rule out the non-specific dephosphorylation of CRY2 in this experiment, this possibility is unlikely because inhibitors of multiple phosphatases were included in all our co-IP reactions. A more plausible explanation is that PPK1 has a higher affinity for unphosphorylated CRY2 relative to phosphorylated CRY2. Figure 2c shows that light treatment results in a dramatic increase in the relative amount of slow-migrating phosphorylated CRY2 and a concomitant decrease in the relative level of the faster-migrating unphosphorylated CRY2 in the presence of PPK1 (Fig. 2c,d, Input lanes). However, the relative levels of CRY2 co-precipitated by PPK1, primarily in the form of the faster-migrating unphosphorylated CRY2, did not show such dramatic decreases in response to light (Fig. 2c,d, IP lanes). The decrease of the unphosphorylated CRY2 in light-treated cells and the lower affinity of the phosphorylated CRY2 for PPK1 appears to explain the lack of overall increase of the CRY2–PPK1 complex detected in the HEK293T cells in response to light. In keeping with this possibility, the catalytically inactive PPK1^D267N^ mutant (also see later) co-precipitated equal amounts of CRY2 regardless of light treatment (Fig. 2d, comparing the corresponding Input and Flag-IP lanes). However, the light-dependent change in the ratio of phosphorylated and unphosphorylated CRY2 and the differential affinity of PPK1 for phosphorylated and unphosphorylated CRY2 do not seem to fully explain why a blue light-dependent increase in the CRY2–PPK1 complex was detected in plants (Fig. 2a) but not in the heterologous human cells (Fig. 2c,d). This discrepancy may be explained by the non-native conditions in human cells, in which *Arabidopsis* CRY2 may experience an accelerated thermo relaxation or dark reversion from its photoexcited state, the ‘wrong' stoichiometry of CRY2 and PPK1, or a lack of other plant proteins required for the blue light-enhanced CRY2–PPK1 interaction. To verify the blue light-enhanced PPK1–CRY2 interaction, we analysed blue light effects on the formation of CRY2–PPK1 complex in plants using a co-IP assay. Figure 2e shows that endogenous CRY2 immunoprecipitated by the anti-CRY2 antibody co-precipitated apparently more PPK1 from light-treated seedlings than that from etiolated seedlings (Fig. 2e). This result confirms that blue light stimulates formation of the CRY2–PPK1 complex in plants. However, this experiment could not clarify whether PPKs preferentially interact with unphosphorylated CRY2 in plants, because phosphorylated CRY2 is rapidly ubiquitinated and degraded in etiolated seedlings exposed to blue light^8^,^16^,^17^,^18^. On the basis of the observations that only photoexcited CRY2 is phosphorylated, that blue light stimulates formation of the PPK–CRY2 complex in plants, and that the non-catalytic PPKC domain interacts with CRY2 in the blue light-dependent manner in yeast cells, we conclude that PPKs preferentially interact with photoexcited CRY2. We also hypothesize that PPKs may have higher affinity for photoexcited and unphosphorylated CRY2 relative to the photoexcited but phosphorylated CRY2. This hypothesis is consistent with the common expectation of a typical enzyme described by the Michaelis–Menten model but it remains to be further tested.

### PPKs catalyse blue-light-dependent phosphorylation of CRY2

**Figure 3:**
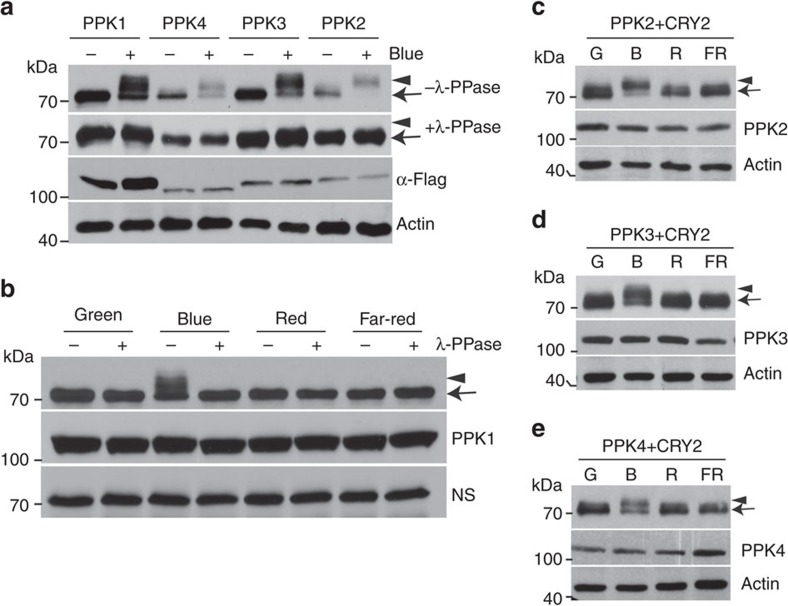
Immunoblot assays showing PPK-dependent catalysis of the blue light-dependent phosphorylation of CRY2. (**a**) HEK293T cells co-expressing *CMV::Myc-CRY2* and individual *CMV::Flag-GFP-PPK* (*PPK1* to *PPK4*) were grown in the dark without blue light treatment (Blue −) or exposed to blue light (Blue +, 30 μmol m^−2^ s^−1^) for 2 h. Lysates were treated without (−λ-PPase) or with λ-PPase (+λ-PPase) for 30 min, and analysed by immunoblots probed with the anti-CRY2 (top two panels) or anti-Flag antibody (α-Flag) respectively. (**b**) HEK293T cells co-expressing *CMV::Myc-CRY2* and *CMV::Flag-GFP-PPK1* were exposed to green (30 μmol m^−2^ s^−1^), blue (30 μmol m^−2^ s^−1^), red (30 μmol m^−2^ s^−1^) or far-red light (5 μmol m^−2^ s^−1^) for 60 min, and analysed by immunoblots as in **a**. (**c**–**e**) Same as (b), except that *CMV::Myc-CRY2* was co-transfected with *CMV::Flag-GFP-PPK2* (**c**), *CMV::Flag-GFP-PPK3* (**d**) or *CMV::Flag-GFP-PPK4* (**e**).

**Figure 4:**
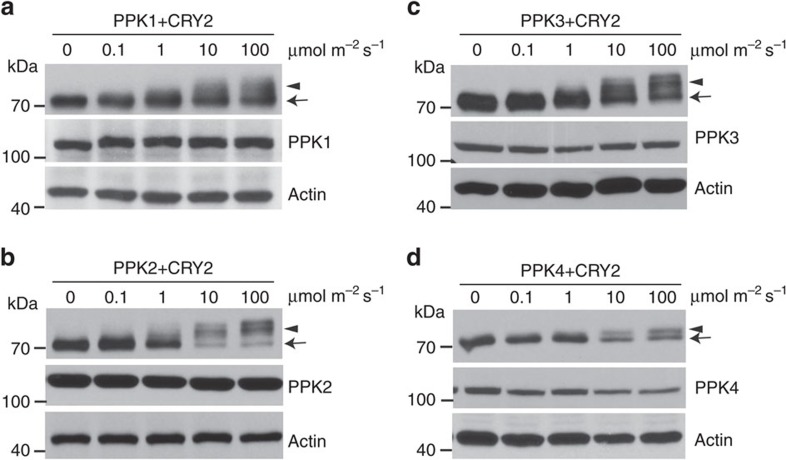
The fluence-rate response of the light-dependent phosphorylation of CRY2 catalysed by PPKs. Immunoblot assays showing the fluence-rate response of the blue light-dependent phosphorylation of CRY2 catalysed by PPK1 (**a**), PPK2 (**b**), PPK3 (**c**) and PPK4 (**d**) in HEK293T cells. Cells co-transfected to express *CMV::Myc-CRY2* and indicated individual *CMV::Flag-GFP-PPKs* were exposed to the different fluence rates of blue light (0–100 μmol m^−2^ s^−1^) for 30 min. CRY2 and PPK were detected by immunoblots probed with anti-Myc or anti-Flag antibodies, respectively. Arrowheads and arrows indicate phosphorylated CRY2 (upshift bands) and unphosphorylated CRY2, respectively.

**Figure 5:**
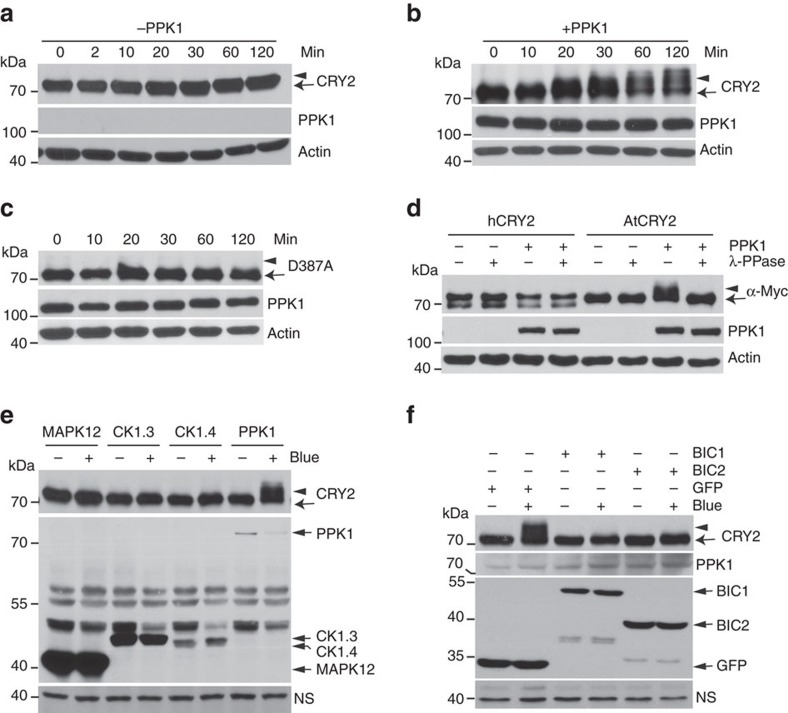
Immunoblot analyses of the substrate specificity of PPKs. (**a**-**c**) HEK293T cells expressing *CMV::Myc-CRY2* (**a**,**b**) or chromophore-deficient *CMV::Myc-CRY2*^*D387A*^ (c) in the absence (a) or presence of the co-expressed *CMV::Flag-GFP-PPK1* (**b**,**c**) were exposed to blue light (30 μmol m^−2^ s^−1^) for the indicated time. CRY2 and PPK1 were detected by the anti-CRY2 or anti-Flag antibodies, respectively. (**d**) HEK293T cells expressing *CMV::Myc-hCRY2* or *CMV::Myc-AtCRY2* in the absence (PPK1−) or presence of *CMV::Flag-GFP-PPK1* (PPK1+) were exposed to blue light (30 μmol m^−2^ s^−1^) for 2 h. Lysates were treated with (λ-PPase +) or without λ-PPase (λ-PPase −), and analysed by immunoblots probed with the anti-Myc (α-Myc) or anti-Flag (PPK1) antibody, respectively. (**e**) HEK293T cells co-expressing CRY2 and PPK1 or indicated kinases are analysed as in **a**. (**f**) HEK293T cells co-expressing *CMV::Myc-CRY2* and *CMV::Flag-PPK1* in the presence of *CMV::GFP-BIC1*, *CMV::GFP-GFP-BIC2*, or *CMV::GFP* were kept in the dark (Blue −) or exposed to blue light (30 μmol m^−2^ s^−1^, Blue +) for 1 h, and analysed by immunoblots probed with anti-CRY2 (CRY2), anti-Flag (PPK1) or anti-GFP (BIC1, BIC2 or GFP) antibody, respectively.

We next investigated the catalytic characteristics of PPKs, especially the light dependency and substrate specificity. In this experiment, we analysed PPK-dependent CRY2 phosphorylation in HEK293T cells co-expressing CRY2 and individual PPKs, using an electrophoretic-mobility-shift immunoblot assay^8^,^16^,^17^,^18^. Figure 3a shows that all four PPKs exhibited enzymatic activity to generate a blue light-dependent and phosphatase-sensitive slower-migrating form of CRY2, confirming that all PPKs are capable of phosphorylating CRY2. Consistent with CRY2 phosphorylation in plants^16^, PPK-catalysed CRY2 phosphorylation in HEK293T cells was dependent on the specific wavelength of blue light (Fig. 3b–e), the fluence rate of blue light (Fig. 4), and total fluence or time of blue-light exposure (Fig. 5a,b, Supplementary Fig. 6). In addition, the blue light-dependent CRY2 phosphorylation is also dependent on the dosage of PPKs (Supplementary Fig. 7).

If PPKs are the authentic protein kinase catalysing blue light-dependent phosphorylation of photoexcited CRY2 *in vivo*, they should catalyse blue light-dependent phosphorylation of the CRY2 holoprotein but not the CRY2 apoprotein. Indeed, PPK1 failed to phosphorylate the D387A mutant of CRY2 (CRY2^D387A^) that is chromophore-deficient and physiologically inactive (Fig. 5c)^11^. No electrophoretic mobility shift was detected for human CRY2 (Fig. 5d) indicating that hCRY2 is not a substrate of *Arabidopsis* PPK1. Importantly, CRY2 was not phosphorylated by a MAP kinase (MAPK12) that was included as the negative control (Fig. 5e). We also examined phosphorylation of CRY2 co-expressed with two isomers of *Arabidopsis* casein kinase 1, CK1.3 and CK1.4, in HEK293T cells, because CK1.3 and CK1.4 were previously reported to phosphorylate CRY2 (ref. 27). In contrast to PPK1, co-expression of CK1.3 or CK1.4 with CRY2 resulted in no blue light-dependent electrophoretic mobility shift of CRY2 (Fig. 5e). Although the phosphorylation of some residues of CRY2 may not lead to an electrophoretic mobility shift^18^, this does not explain the lack of a CK1-dependent electrophoretic mobility shift of CRY2 (see later). Because the recently reported blue-light inhibitors of cryptochromes, BIC1 and BIC2, suppress CRY2 phosphorylation in plants^8^, we also tested whether BICs might inhibit CRY2 phosphorylation in HEK293T cells. As expected for an authentic CRY2 kinase, the catalytic activity of PPK1 phosphorylating CRY2 is suppressed by BIC1 or BIC2 expression in HEK293T cells (Fig. 5f).

**Figure 6:**
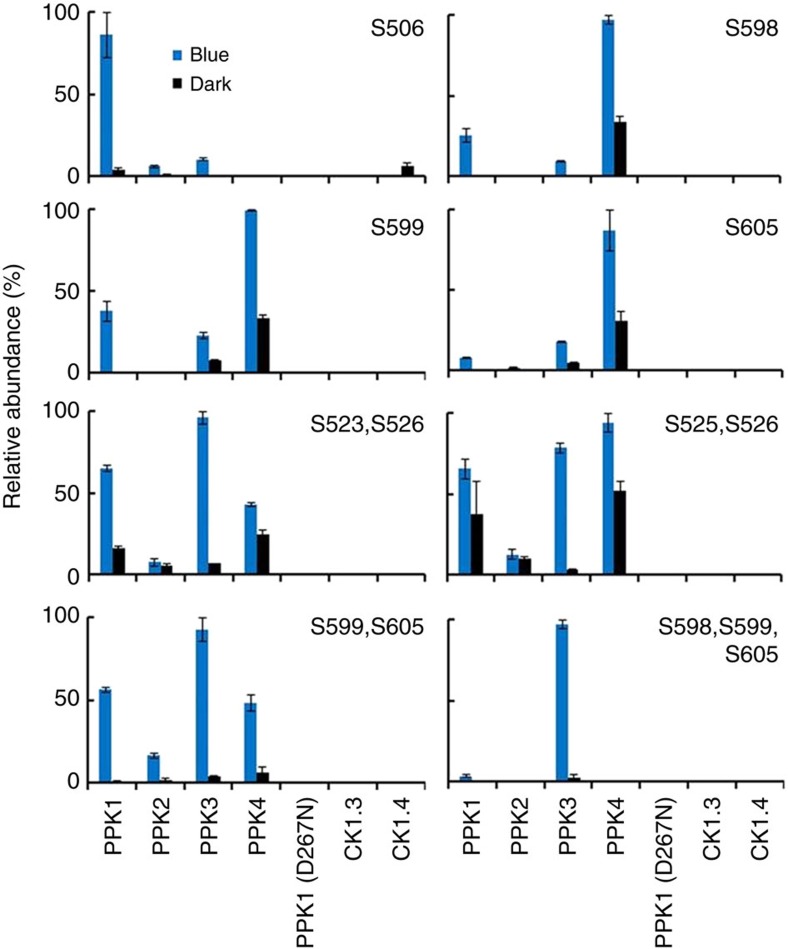
MS analyses of the phosphosites of PPK-catalysed phosphorylation of CRY2. HEK293T cells co-expressing CRY2 and a Flag-tagged PPK indicated, CK1.3, CK1.4, or the catalytically inactive PPK1^D267N^ mutant (PPK1-D267N) were exposed to blue light (30 μmol m^−2^ s^−1^, 120 min) (blue bars) or kept in the dark (black bars). Phosphopeptides of CRY2 purified from individual samples were analysed by the label-free quantitative MS, and the most abundant phosphopeptides detected in this experiment are shown. The lack of a bar indicates that the peptide/protein was not detected. The intensity of each phosphopeptide was quantified by PRM, analysed by the Skyline software, normalized against total ion current, and converted to relative abundance as described in Fig. 1a. Three biological replicates are analysed and the standard deviations are shown (*n*=3).

Another important expectation for an authentic CRY2 kinase is that it should phosphorylate CRY2 on the same residues known to undergo phosphorylation in plants (Fig. 1). To test this hypothesis, we compared the phosphosites of CRY2 purified from plants with the phosphosites of CRY2 purified from the HEK293T cells co-expressing each of the four PPKs. We included a catalytically inactive PPK1 (PPK1^D267N^), as the negative control (see later). We also analysed the phosphosites of CRY2 purified from HEK293T cells co-expressing CK1.3 or CK1.4. Figure 6 shows most phosphopeptides detected in GFP–CRY2 purified from HEK293T cells co-expressing PPKs overlap with the phosphopeptides detected in GFP–CRY2 purified from plants (Figs 1 and 6). As expected, the combined activities of the four PPKs resulted in phosphorylation of CRY2 on all seven major phosphosites of CRY2 detected *in vivo*, including S506, S523, S525, S526, S598, S599 and S605 (Fig. 6). Intriguingly, individual PPKs exhibited clearly different phosphosite preferences (Fig. 6). In contrast, no phosphopeptide was detected in GFP-CRY2 purified from HEK293T cells co-expressing CK1.3 or CK1.4 (Fig. 6). Although CK1.3 and CK1.4 are previously reported to phosphorylate residues S587 and T603 of CRY2 (ref. 27), phosphorylation on S587 or T603 was not detected in GFP–CRY2 purified from plants (Fig. 1) or in GFP-CRY2 purified from HEK293T cells co-expressing CK1.3 or CK1.4 (Fig. 6). Although, we cannot rule out a possibility that phosphopeptides encompassing S587 might be difficult to detect in our MS analyses, it seems unlikely that phosphorylation of T603 would escape detection. Because the residue T603 is present in many of the phosphopeptides we detected, including singly phosphorylated peptides modified on the adjacent T604 (Fig. 1b), doubly phosphorylated peptides modified on S599 and S605 (Figs 1 and 6), and the triply phosphorylated peptide modified on S598, S599 and S605 (Figs 1 and 6), the fact that we did not observe T603 phosphorylation in CRY2 purified from plants or HEK293T cells co-expressing CK1.3 or CK1.4 (Figs 1 and 6) is most likely explained by the absence of CRY2 phosphorylation on T603 under the described conditions. Given the lack of CK1.3- or CK1.4-dependent phosphorylation in all three experiments (Figs 1, 5 and 6), our results indicate that CK1.3 and CK1.4 are not likely to be the major protein kinases catalysing blue light-dependent phosphorylation of CRY2.

**Figure 7:**
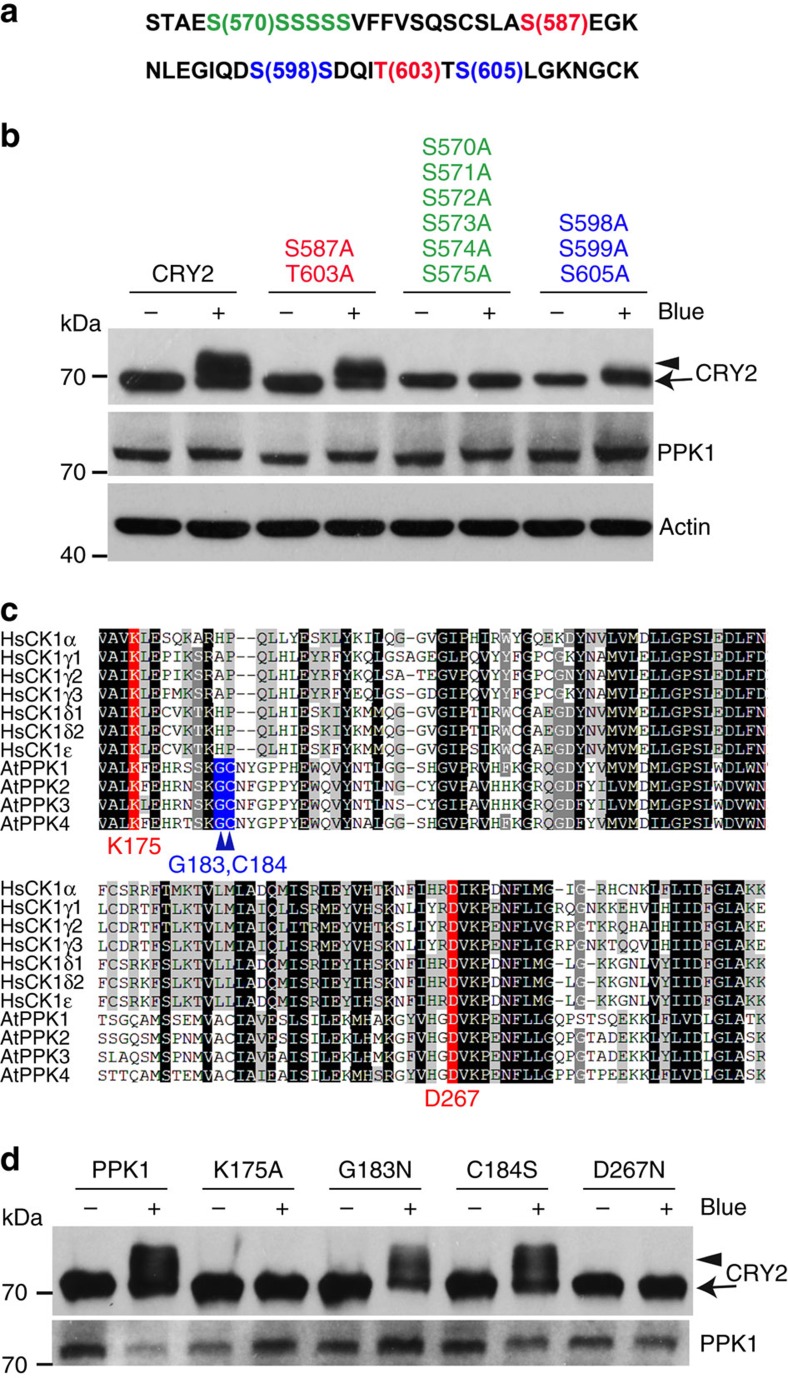
Mutation analyses of PPK1-catalysed and blue light-dependent phosphorylation of CRY2. (**a**) A partial amino acid sequence in CRY2, in which the phosphosites subject to site-specific mutations are highlighted by colours. (**b**) Immunoblot showing PPK1-catalysed phosphorylation of CRY2 and three indicated CRY2 mutants corresponding to the phosphosites shown in **a**, including CRY2^S587A/T603A^, which was the proposed CK1.3 and CK1.4 phosphorylation sites, CRY2^S570A/S571A/S572A/S573A/S574A/S575A^, which contains mutations in the serine cluster (S570–S575), and CRY2^S598A/S599A/S605A^, which contains mutations in three serine residues (S598, S599 and S605) that are the major phosphosites of CRY2 detected (Fig. 1b). Cells transfected to co-express *CMV::Myc-CRY2* or *CRY2* mutants and *CMV::Flag-PPK1* were kept in the dark (Blue −) or exposed to the blue light (Blue +; 30 μmol m^−2^ s^−1^) for 60 min. CRY2 and PPK1 were detected by immunoblot probed with anti-Myc or anti-Flag antibodies, respectively. (**c**) Amino acid sequence alignment of the kinase domain in *Arabidopsis* PPKs and human CK1s. The two evolutionarily conserved residues (K and D, including K175 and D267 of PPK1) are labelled in red; the other two residues that are conserved only in *Arabidopsis* PPKs (G and C, including G183 and C184 of PPK1) but not in CK1 are labelled in blue. (**d**) HEK293T cells co-expressing *CMV::Myc-CRY2* and *CMV::Flag-PPK1* or the *CMV::Flag-PPK1* mutants were treated with blue light and analysed by immunoblot probed by anti-CRY2 or anti-Flag, respectively.

To further verify the CRY2 phosphosites detected by the MS analyses, we examined the ability of PPK1 to phosphorylate two different CRY2 mutants. One mutant contains serine-to-alanine mutations of three major phosphosites (S598A/S599A/S605A), and another mutant contains serine-to-alanine mutations of six putative minor phosphosites of the serine cluster (S570A/S571A/S572A/S573A/S574A/S575A) (Fig. 7a,b). We have previously shown that the serine-to-alanine mutation of these phosphosites caused progressively reduced phosphorylation and physiological activity of CRY2 *in vivo*^18^. As expected, neither of these two CRY2 mutants were phosphorylated by PPK1 (Fig. 7a,b). As a control, we also prepared and tested a third mutant of CRY2 (S587A/T603A), which contains the serine-to-alanine and threonine-to-alanine mutations in the reported CK1.3 and CK1.4 phosphosites, S587 and T603. But the S587A/T603A mutants of CRY2 were phosphorylated normally by PPK1 (Fig. 7b). Although lack of protein phosphorylation in any phosphosite mutation may result from indirect effects of structural changes or the direct effect of the loss of phosphosites, the independent lines of evidence described above support the hypothesis that PPKs are the major protein kinases that catalyse the blue light-dependent phosphorylation of CRY2 in plants.

Although PPKs form an independent phylogenetic clade distinct from casein kinase I (Supplementary Fig. 4)^27^,^31^,^32^, the kinase domains of PPKs exhibit ∼40% amino acid sequence identity to that of CK1s (Supplementary Fig. 5b). The modelled three-dimensional structure of the kinase domain of PPK1 resembles that of a canonical casein kinase 1 (Supplementary Fig. 8)^34^,^35^,^36^,^37^. We prepared two PPK1 mutants at residues K175A and D267N, which are conserved in both PPKs and CK1s and are located at the positions equivalent to the known catalytic residues in the ATP- and substrate-binding pocket of CK1s (Fig. 7c, Supplementary Fig. 8)^38^,^39^. Figure 7d shows that mutation at either K175A or D267N eliminated the catalytic activity of PPK1. In contrast, mutations in two nearby residues within the catalytic pocket of PPK1, G183 and C184, that are conserved in PPKs but not in CK1 (Fig. 7c, Supplementary Fig. 8), did not significantly affect the PPK1 activity (Fig. 7d). These results suggest that, despite their overall structural and functional divergences, PPKs and CK1s share the similar catalytic structure and mechanism.

### Blue light-dependent phosphorylation of CRY2 requires *PPKs*

**Figure 8:**
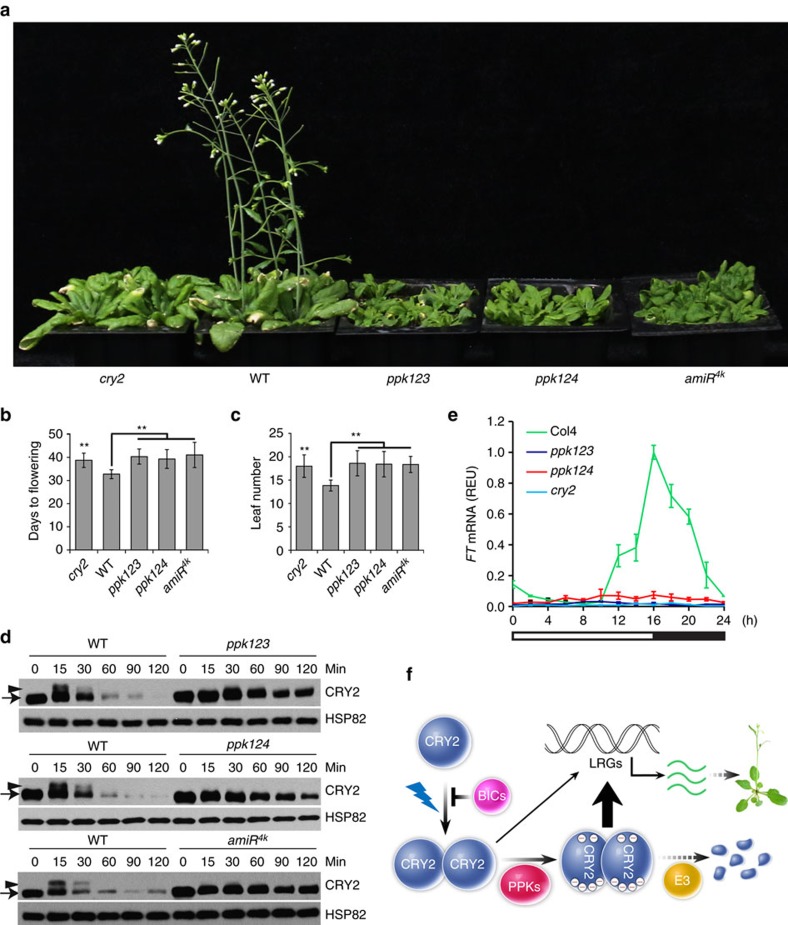
Genetics analyses of PPK-dependent CRY2 phosphorylation. (**a**) 27-day-old plants of indicted genotypes, including the *ppk123* and *ppk124* mutants, and a *amiR*^*4k*^-expressing line, grown in LD photoperiods (16 h light, 8 h dark) are shown. (**b**,**c**) Flowering time of the indicated genotypes measured as ‘leaf number' (**b**) or ‘Days to flowering' (**c**), with the s.d. shown (*n*>20). ** in (**b**,**c**) indicate *P*<0.01. (**d**) Immunoblot showing the lack of blue light-dependent CRY2 phosphorylation and degradation in the *ppk123* and *ppk124* mutants, and an *amiR*^*4k*^-expressing line. Etiolated seedlings of the indicated genotypes were exposed to blue light (5 μmol m^−2^ s^−1^) for the indicated time (0–120 min) before sample collection. Proteins were analysed by immunoblot probed with the anti-CRY2 (CRY2) or anti-HSP82 (HSP82) antibody, respectively. (**e**) Relative abundance of *FT* mRNA in 12-day-old plants of indicated genotypes grown in LD photoperiods. Relative expression unit (REU) was calculated by normalization to the reference gene *IPP2* (AT3G02780). S.d. (*n*=3) are shown. (**f**) A model depicting the molecular basis of the blue light-dependent phosphorylation and function of CRY2. Photoexcited CRY2 undergoes homodimerization and PPK-catalysed phosphorylation. Light regulation of light regulated genes (LRGs, such as *FT*) is mediated by the partially active unphosphorylated CRY2 dimer (thin arrow) and the fully active phosphorylated CRY2 dimer (thick arrow). Phosphorylated CRY2 is ubiquitinated by E3 ligases and degraded by the 26S proteasome.

We next investigated the roles of PPKs in plant development and how PPKs affect the function of CRY2 in plants. Because the *PPK* genes are known to function redundantly^29^,^30^, we prepared and analysed *amiR*^*4k*^ transgenic lines that express multiple artificial microRNAs to suppress messenger RNA (mRNA) expressions of all four *PPK* genes (Fig. 8, Supplementary Figs 9–11), as well as *ppk123* and *ppk124* triple mutants (Fig. 8)^40^. Similar to the *cry2* mutant^4^, the *ppk123* and *ppk124* triple mutants and independent *amiR*^*4k*^ lines exhibited delayed flowering in long-day photoperiods (Fig. 8a–c, Supplementary Fig. 10), which is consistent with the long-standing hypothesis that blue light-dependent phosphorylation of CRY2 enhances its activity^8^,^16^,^17^,^18^. The mRNA expression of the *FT* gene, a key activator of floral initiation regulated by CRY2 via both direct and indirect transcriptional activation mechanisms^11^,^41^,^42^,^43^, was reduced in the *ppk123, ppk124* plants (Fig. 8e). These observations can be at least partially explained by the reduced CRY2 phosphorylation and activity, although other PPK substrates might also contribute to the phenotype of reduced *FT* mRNA expression and delayed flowering of the *ppk* mutants. Importantly, the blue light-dependent phosphorylation of CRY2 is clearly impaired in the *ppk123* mutant, the *ppk124* mutant, and the *amiR*^*4k*^ transgenic lines (Fig. 8d). Consistent with CRY2 phosphorylation being required for the ubiquitination and degradation of CRY2 (refs 8, 16, 17, 18), the blue light-dependent degradation of CRY2 was suppressed in all three mutant lines (Fig. 8e). On the basis of these results, we propose a hypothetical model to explain the molecular mechanism underlying blue light-dependent CRY2 phosphorylation, regulation and function (Fig. 8f). According to this model, PPKs interact with photoexcited CRY2 dimer to phosphorylate multiple phosphosites in both the PHR and the CCE domains of CRY2 resulting in conformational changes that increase the CRY2-mediated light responses. The PPK-dependent phosphorylation of CRY2 also stimulates ubiquitination and degradation of the photoreceptor.

Given that cryptochromes mediate blue light inhibition of hypocotyl growth and our previous results that progressive mutations of multiple phosphosites of CRY2 progressively reduced phosphorylation of CRY2 and its activity mediating blue light inhibition of hypocotyl growth^18^, one may expect that the *ppk* mutants and *amiR*^*4k*^ lines should exhibit reduced photosensitivity and increased hypocotyl growth in light. Surprisingly, in contrast to what we expected, the *ppk* mutants and *amiR*^*4k*^ lines exhibited photo-hypersensitivity and shorter hypocotyls in both blue light and red light conditions tested (Supplementary Fig. 11). This result demonstrates that, in addition to CRY2, PPKs must have other substrates and some of those substrates act genetically downstream from photoreceptors to regulate light inhibition of hypocotyl elongation. Indeed, PPKs are reported to interact and phosphorylate the phytochrome signalling protein PIF3 in an accompanying paper^40^. This result explains the photo-hypersensitive phenotype of the loss-of-function *ppk* mutants and *amiR*^*4k*^ knock-down lines in response to red light. PPKs may also interact and phosphorylate other related PIF proteins in addition to PIF3. The fact that PIF4 and PIF5 are known to interact with cryptochromes and act in blue light regulation of hypocotyl growth^44^,^45^ may partially explain the photo-hypersensitive phenotype of the *ppk* mutants and the *amiR*^*4k*^ knock-down lines in response to blue light. Furthermore, given that PPKs are known to physically associate with the evening complex (ELF3–ELF4–LUX) of the circadian clock^30^, phosphorylation of components or regulators of the circadian oscillator may also explain the wavelength-independent photo-hypersensitive hypocotyl growth of the *ppk* mutants and the *amiR*^*4k*^ knock-down lines.

## Discussion

It has been proposed that cryptochromes evolved independently from common ancestral DNA photolyases in major evolutionary lineages, such as plants and animals^1^,^2^. Consistent with this hypothesis, we showed that PPKs, but not plant orthologs of the protein kinases catalysing phosphorylation of the mammalian cryptochromes, such as AMPK (ref. 46), CK1 (ref. 47) and GSK3β (ref. 24), are the major cryptochrome kinases in *Arabidopsis*. It is not fully understood why plants retain four closely related and similarly expressed PPKs to phosphorylate CRY2. The apparently larger number of phosphosites detected in *Arabidopsis* CRY2 in comparison to the more limited number of phosphosites detected in mammalian cryptochromes^24^,^46^,^47^ and different phosphosite preferences of different PPKs (Fig. 6) may provide a partial explanation. However, a more compelling proposition may be that multiple PPKs are needed to coordinately regulate multiple photoregulatory proteins of plant photoresponses and the circadian oscillator that are often associated in related protein assemblies^14^,^40^,^44^,^45^,^48^,^49^. The discoveries that PPKs interact with and phosphorylate CRY2 and PIF3 are consistent with this hypothesis. We speculate that PPKs may act as the photoregulatory hubs that catalyse phosphorylation of diverse protein substrates associated with different light signalling and timing apparatus to coordinate light- and time-dependent control of plant growth and development. Finally, given that red light and blue light are the two major wavelengths utilized by photosynthesis and developmental regulation, the discovery of direct roles for PPKs in modifying and regulating key components of both cryptochrome and phytochrome pathways reveals a previously unknown mechanism underlying coordination of the functions of cryptochromes and phytochromes in response to solar radiation in nature.

## Methods

### Plant materials and growth conditions

All *Arabidopsis* lines used in this work are of the Columbia (Col) accession. The *ppk123, ppk124 and pif1345* mutants are gifts from Dr Peter H. Quail. The *ppk* single mutants, *ppk1* (GABI_756G08), *ppk2* (SALK_026482), *ppk3* (SALK_000758) and *ppk4* (SALK_017102C), were used for triple mutants preparation. To prepare PPK artificial microRNA (amiRNA) knock-down lines, the amiRNAs targeting *PPKs* were designed online (http://wmd3.weigelworld.org/cgi-bin/webapp.cgi), using the WMD3 Web microRNA Designer^50^. The gene-silencing efficiency of the amiRNA candidates was tested in plants (described in ‘Tobacco transient expression'), the most effective microRNAs were selected for each *PPK* gene. The amiRNA precursor was derived from pRS300 using overlapping PCR. The amiRNA targeting *PPK1* and *PPK4* were cloned into Ti plasmid (*pDT1B*). The amiRNAs targeting *PPK2* and *PPK3* were cloned into another Ti plasmid (*pDT1H*). Ti plasmid *pDT1B* (Basta) and *pDT1H* (hygromycin) were modified from *pCambia3301*, which possess two expression cassettes. The recombinant plasmid, *pDT1B-amiPPK1-amiPPK4* and *pDT1H-amiPPK2-amiPPK3*, were co-transformed into the *Arabidopsis* (Col) using the standard floral-dip method^51^. The transgenic T1 populations were screened on MS agar media containing 25 mg l^−1^ Hygromycin and 25 mg l^−1^ Basta. To generate *GFP-PPKs* lines, the coding sequences of PPKs were PCR-amplified and cloned into a Ti plasmid (*pFGFP*) modified from *pCambia3301* by the In-Fusion cloning method, resulting in expression of *GFP-PPKs* driven by the *ACTIN2* promoter (*pACT2::GFP-PPKs*). Ti plasmids expressing recombinant proteins were introduced into *rdr6-11* allele, which suppresses gene silencing, by the floral-dip method^51^. The transgenic T1 populations were screened on compound soil sub-irrigated with the Basta solution. Plants were grown in walk-in growth chambers at 22 °C, 65% relative humidity under cool white fluorescent tubes. Long-day (LD) photoperiod is defined as 16 h light/8 h dark. Light-emitting diode was used to obtain monochromatic blue light (peak 450 nm; half-bandwidth of 20 nm), red light (peak 660 nm; half-bandwidth of 20 nm) or far-red light (peak 730 nm; half-bandwidth of 20 nm).

### Yeast two-hybrid assays

The prey vector *pGADT7* expressing PPK or PPKC fused to the GAL4 activation domain and the Bait vector *pBridge* expressing CRY2 fused to the GAL4 DNA binding domain were used. The pairs of plasmids were co-transformed into yeast strain AH109. Colonies were selected on plates (SD-LW). After culture in SD-LW medium for 16 h (Dark), transformants were subcultured into fresh SD-LW medium and kept in dark or irradiated with blue light (30 mol m^−2^ s^−1^). β-galactosidase activity was measured using chlorophenol red-β-D-galactopyranoside as substrate and Miller Units were calculated according to the Clontech Yeast Protocols Handbook.

### Tobacco transient expression

To test the gene-silencing efficiency of those designed amiRNAs, the Ti plasmids expressing 4 × Myc-PPKs was co-transformed with the plasmids expressing the corresponding amiRNA or the empty plasmid into *Nicotiana benthamiana* leaves. The efficiency of amiRNA was evaluated by examination of the PPK expressed from the epitope-tagged PPK-expressing plasmids co-transfected with the amiRNA-expressing plasmids, using immunoblot.

For co-localization assay of CRY2 and PPKs proteins, the recombinant Ti plasmid (described above) expressing CRY2-mCherry and GFP–PPKs were first transformed into *Agrobacterium* strain AGL0 respectively, and then co-transfected with *Nicotiana benthamiana* leaves. Briefly, overnight cultures of *Agrobacteria* were collected by centrifugation, the pellets were suspended in MES buffer (10 mM MES pH 5.6, 10 mM MgCl_2_ and 150 μM acetosyringone) to 1.5 OD600. Transfected *Agrobacteria* were mixed and infiltrated into healthy leaves of three-week-old *Nicotiana benthamiana* using a 1 ml syringe. After infiltration and kept in the dark overnight, plants were then grown in the growth chamber with continuous white light for 2 days before harvesting for immunoblot assays or microscopic observation. mCherry and GFP signals were detected using the confocal laser scanning microscope (LSM 880, Carl Zeiss).

### RNA extraction and qPCR

Total RNA was extracted from *Arabidopsis* seedlings using RNeasy Plant Mini Kit (QIAGEN). After removing the genomic DNA, 1 μg of total RNA was used for single-strand complementary DNA (cDNA) synthesis using Superscript III reverse transcriptase (Invitrogen) with oligo-dT primers following the manufacturer's protocol. Quantitative PCR reactions were performed with gene specific primers and the SYBR Premix Ex Taq (TaKaRa) on a Mx3005P Real-Time PCR System (Stratagene). The qPCR signals were normalized to that of the reference gene *IPP2* (AT3G02780) using the ΔCT method. The PCR primers used in this experiment are summarized in Supplementary Table 2.

### The HEK293T protein expression and kinase assay system

HEK293T cells were routinely cultured in Dulbecco's modified Eagle's medium (DMEM) supplemented with 10% (v/v) FBS, 100 IU penicillin and 100 mg l^−1^ streptomycin in humidified 5% (v/v) CO_2_ in air, at 37 °C. The coding sequences of *Arabidopsis* CRY2, BICs, PPKs and other kinases were cloned into the *pCMV* or the modified *pEGFP-N1* vectors (Clontech) by fusing in frame to the DNA sequence encoding the Myc, GFP, Flag, or Flag-GFP epitope tags. Flag-PPK1 appears to have a generally higher catalytic activity compared to the Flag-GFP-PPK1 fusion protein. HEK293T cells were seeded at a density of ∼3 × 10^5^ cells per well of a six-well plate and transfected using a calcium phosphate precipitation protocol. Briefly, plasmid DNA (1–2 μg) was mixed with 2.5 M CaCl_2_ (1/20 of total volume) and 2 × HeBS (250 mM NaCl, 10 mM KCl, 1.5 mM Na_2_HPO_4_, 12 mM Dextrose and 50 mM HEPES pH 7.5, adjusted the pH of the final solution to 7.05), and kept at room temperature for 5 min before applying to host cells. The medium was aspirated from each well, the DNA mixture was added slowly into wells without disturbing the cells on the bottom, and the plates were rotated gently to allow the solution to coat the whole well. 2 ml fresh media containing 25 μM chloroquine was added to each well, the plates were placed in a 37 °C incubator overnight, and the culture medium was changed with 2 ml fresh medium without chloroquine per well. The cells were usually collected 36–48 h after transfection.

### Immunoblot and co-immunoprecipitation assays

To prepare the crude protein extracts for immunoblot analysis, the seedlings were ground in liquid N_2_ and boiled in Protein Extraction Buffer (120 mM Tris–HCl pH 6.8; 100 mM EDTA; 4% SDS; 10% β-ME; 5% Glycerol and 0.01% Bromophenol blue) for 10 min. The protein samples were separated by 10% SDS–PAGE and transferred to Immobilon NC Transfer Membrane (HATF00010, Millipore).

For co-IP experiments using the transfected HEK293T cells as starting materials, the cells were washed once with PBS and lysed in 3 × volumes of 1% Brij buffer (1% Brij-35, 50 mM Tris–HCl pH 8.0, 150 mM NaCl, 1 mM PMSF, 1 × Protease inhibitor cocktail and 1 × PhosSTOP inhibitor cocktail (Roche)). Cell lysates were placed on ice for 20 min, quickly frozen in liquid N_2_ once, and kept on ice for 20–30 min. After centrifuging at 14,000*g* for 10 min at 4 °C, the supernatant was mixed with 20 μl affinity beads (FLAG M2 beads, sigma, F2426), incubated with rotation at 4 °C for 2 h. Beads were pelleted and washed four times with ice-cold 1% Brij buffer (without PMSF and Protease inhibitor cocktail). The bound proteins were eluted from the beads with 2 × SDS–PAGE Sample Buffer by heating at 100 °C for 4 min. Protein samples were separated by 10% SDS–PAGE and transferred to Immobilon NC Transfer Membrane (HATF00010, Millipore).

For co-IP experiments using *Arabidopsis* as starting materials, 2 g plant tissues were ground in liquid N_2_ and homogenized in IP buffer (50 mM Tris–HCl pH 7.5, 150 mM NaCl, 1% Triton X-100, 1 mM PMSF, 1 mM EDTA, 20 mM NaF and 1 × Protease inhibitor cocktail). After centrifuging at 14,000*g* for 10 min at 4 °C, the supernatant for each sample was mixed with 40 μl Protein A/G Agarose beads and 20 μl anti-CRY2 antibodies and rotated at 4 °C for 2 h. Then beads were pelleted and washed four times with ice-cold IP buffer (without protease inhibitors). The bound proteins were eluted from the beads with 2 × SDS–PAGE sample buffer by heating at 100 °C for 4 min, and analysed by immunoblot. The membrane was stained with Ponceau S red and blocked with 5% skimmed milk in 1 × PBST (137 mM NaCl, 2.7 mM KCl, 4.3 mM Na_2_HPO_4_, 1.4 mM KH_2_PO_4_ and 0.3% Tween-20, pH 7.4) solution. After probing with the primary and secondary antibodies, the membrane was incubated in the buffer supplied by Super Signal West Pico Chemiluminescent substrate kit (Pierce) to detect signals derived from antibodies. The primary antibodies used in this study are: anti-CRY2 (1:3,000)^8^, anti-HSP82 (1: 8,000, AbM51099-31-PU, BGI), anti-FLAG (1:1,500, F3165, Sigma) and anti-Myc (1:3,000, 05-724, Millipore). Different immunoblots are not directly comparable, because the exposure time of ECL cannot be precisely controlled between different experiments. Original blot images are provided in Supplementary Fig. 12.

### Confocal microscopy

Seeds of transgenic *Arabidopsis* expressing GFP-PPKs were surface sterilized and sowed on the Murashige and Skoog (MS) plates supplemented with 3% (w/v) sucrose. After stratification in the dark at 4 °C for 3 days, plates were kept in the growth chamber with long-day conditions for 5 days. The seedlings were incubated in PI (Propidium Iodide, Sigma P-4170) solution (10 mg l^−1^) for 2 min, quickly dipped in distilled H_2_O, followed by mounting in H_2_O on glass slides and examined by a confocal laser scanning microscope (LSM 880, Carl Zeiss).

### Sample preparation for mass spectrometry analyses

The GFP-CRY2 fusion protein was purified from seedlings overexpressing GFP-CRY2 by the GFP-trap method^18^. Plant tissues were ground in liquid N_2_ and homogenized in IP buffer (50 mM Tris–HCl pH 7.5, 150 mM NaCl, 1% Triton X-100, 1 mM PMSF, 1 mM EDTA, 20 mM NaF and 1 × Protease inhibitor cocktail). After centrifuging at 14,000*g* for 10 min at 4 °C, the supernatant was mixed with GFP-trap agarose beads (gta-100, Chromotek) and rotated at 4 °C for 2 h. Then beads were pelleted and washed four times with ice-cold IP buffer (without protease inhibitors). The bound proteins were eluted from the beads with 2 × SDS–PAGE sample buffer by heating at 100 °C for 4 min. Protein samples were fractionated by 10% SDS–PAGE and stained with Coomassie Brilliant Blue R-250 (ref. 52). Bands of interest were excised and cut into small pieces. After dehydration, proteins in the gel slice were reduced by DTT (10 mM). IAA (55 mM) was then added to alkylate the reduced sulfydryl. After washing and dehydration with acetonitrile, the proteins were digested with trypsin and subjected to MS analyses^52^,^53^.

### LC–MS/MS

Orbitrap-Fusion-Tribrid mass spectrometer (Thermo Scientific) was used for all MS analyses of this study. Data dependent acquisition (DDA) or parallel reaction monitoring (PRM) approaches was used for phosphopeptide or CRY2-interacting protein quantification. For DDA, peptides were subjected to LC–MS/MS analysis using an EASY-nLC 1000 ultra-high pressure liquid chromatography system (Thermo Scientific) connected to an Orbitrap-Fusion-Tribrid mass spectrometer (Thermo Scientific) with aNanospray Flex ion source. Peptides were separated by a two-step gradient at a flow rate of 300 nl min^−1^: 5–20% solvent B (0.1% acetic acid in ACN) in 56 min; 20–32% solvent B in 16 min. The total analysis time for each sample was 90 min. In survey scans, full-scan MS spectra were acquired by the Orbitrap analyser at a resolution of 120,000, scanning from *m*/*z* 350 to *m*/*z* 1550. AGC target was 2e5 and maximum injection time was 50 ms. MS2 quadrupole selection isolation window was 1.6 *m*/*z*. The top speed mode was selected. MS/MS spectra were detected using Orbitrap analyser at the resolution of 15,000. AGC target was 1.0e5 and the maximum injection time was 160 ms. The normalized HCD energy was set at 35%. For PRM, the mass spectrometry system and the chromatography gradient are described as for DDA. The resolution of MS1 full scan was set to 60,000; AGC target was 2e5 and the maximum injection time is set to 100 ms. Full MS scans (MS1) were followed by multiple PRM scans (MS2) at 60,000 resolution (AGC target 8e4, 200 ms maximum injection time) (Supplementary Table 1 b). Precursors are selected by the quadrupole mass analyser using an isolation window set to 1.2 Da.

### MS data processing

For label free quantification, Raw DDA MS files were processed using MaxQuant (version 1.5.2.8). Database searching was performed using Andromeda which is integrated into the MaxQuant^54^,^55^. CRY2-interacting Proteins and phosphopeptides were identified using a target-decoy approach by searching all MS/MS spectra against a concatenated forward/reversed version of TAIR10_pep_20101214 sequence database and filtering using a false discovery rate (FDR)<0.01 at both the peptide and protein level. Carbamidomethylatino of cysteines was selected as the fixed modification while oxidation (M), acetylation (protein N-term) and phosphorylation (STY) were allowed for variable modifications. Match between runs mode) was enabled with a window of 1 min. The database search was performed with an initial precursor ion tolerance of 20 p.p.m., MS/MS tolerance at 0.02 Da, and two missed cleavages are allowed. Phosphosite localization was assessed using Andromeda algorithm and the phosphopeptide containing the residues with phosphor-probability>0.7 were used for further quantification between dark- and blue light-treated samples. Detection of potential phosphosite was further assessed by the Ascore^56^ and phosphoRS algorithms^57^, as indicated in Fig. 1b (ref. 58). After identification, label-free quantitation of phosphopeptides, as well as interacting proteins was performed with the MaxLFQ algorithm integrated in MaxQuant software suite^59^. MaxQuant and integrated MaxLFQ algorithm were detailed described before^51^,^59^. Briefly, the MaxQuant-MaxFLQ algorithms perform the rational assignment of the razor and common peptides to individual homologous proteins, such as SPAs or PPKs. The algorithm next performs the pair-wise ratio determinations and the least square analyses to correct pair-wise random variations and rescale the entire peptide profiles of individual proteins from the proteome analysed. These analyses eventually give rise to a protein-level quantification metric called Label Free Quantification Intensity (LFQI) for each protein. To eliminate the loading variations among different samples, the intensity of phosphopeptide and the LFQI values of each CRY2-interacting protein were normalized against the input intensity (LFQ intensity of CRY2 bait). Finally, we manually convert the normalized LFQI to the more easily understandable ‘relative abundance' (percentage), using the mean of the most abundant phosphopeptide or protein of the three biological replicates as the denominator.

We used MaxQuant-MaxFLQ to quantify the MS1 peak area for phosphoisoforms that were chromatographically distinct from other phosphoisoforms. For more complex phosphoisoform patterns for which we could not chromatographically separate the isoforms, we acquired data using PRM and quantified at the MS2 level using isoform-specific fragment ions using Skyline. PRM data analyses were performed using Skyline daily (version 3.5)^60^, which is widely used for phosphopeptide quantification^61^,^62^,^63^. The intensity of phosphopeptide was derived from the peak area of specific daughter ion, and all Skyline integrated PRM data analysis were manually checked to validate peak selection. The intensity of the phosphopeptide detected was normalized against the total ion current to correct input variations. Finally, we also manually convert the normalized intensity of phosphopeptide to the more easily understandable ‘relative abundance' (percentage), using the mean of the most abundant phosphopeptide of the three biological replicates as the denominator.

### Data availability

The authors declare that all data supporting the findings of this study are available within the manuscript and its supplementary files or are available from the corresponding authors on request.

## Additional information

**How to cite this article:** Liu, Q. *et al*. Molecular basis for blue light-dependent phosphorylation of *Arabidopsis* cryptochrome 2. *Nat. Commun.* **8,** 15234 doi: 10.1038/ncomms15234 (2017).

**Publisher's note:** Springer Nature remains neutral with regard to jurisdictional claims in published maps and institutional affiliations.

## Supplementary Material

Supplementary Information Supplementary Figures and Supplementary Tables
