# Supplementary material for: Molecular basis for blue light-dependent phosphorylation of Arabidopsis cryptochrome 2
Source: Nat Commun. 2017 May 11;8:15234. doi: 10.1038/ncomms15234 (PMC5437284; doi:10.1038/ncomms15234)
Supplement: Supplementary Information — Supplementary Figures and Supplementary Tables [file ncomms15234-s1.pdf]

Supplementary Table 1

a

Phosphosite	MS Intensity (Blue)	Blue/Dark	P Value
S506	5.0E+08	29.8	6.0E-07
S525	6.0E+07	0.2	1.3E-04
S598	2.0E+07	5.0	1.4E-03
S599	2.5E+07	∞	2.2E-05
S605	1.0E+08	7.8	1.9E-03
S523, S526	1.4E+07	2.3	1.9E-03
S525, S526	3.6E+07	10.5	8.6E-03
S599, S605	1.6E+07	∞	1.9E-02

b

Precursors	z	m/z	Modifications
EAQIMGAAPDEIVADSFEALGANTIK	3	951.7807	1xP(79.966)
EAQIMGAAPDEIVADSFEALGANTIK	3	957.4536	1xP(79.966), 1xO(15.995)
EPGLCPSVSSNDQQVPSAVR	3	736.3324	1xP(79.966)
EPGLCPSVSSNDQQVPSAVR	3	762.9871	2xP(159.932)
EPGLCPSVSSNDQQVPSAVR	3	789.6418	3xP(239.898)
EPGLCPSVSSNDQQVPSAVR	2	1103.9913	1xP(79.966)
EPGLCPSVSSNDQQVPSAVR	2	1143.9730	2xP(159.932)
NLEGIQDSSDQITTSLGK	3	662.6417	1xP(79.966)
NLEGIQDSSDQITTSLGK	3	689.2955	2xP(159.932)
NLEGIQDSSDQITTSLGK	2	1033.4390	2xP(159.932)
NLEGIQDSSDQITTSLGK	2	1073.4210	3xP(239.898)
NLEGIQDSSDQITTSLGKNGCK	3	816.0331	1xP(79.966)

Supplementary Table 1. Identification of the in vivo phosphosites of GFP-CRY2 purified from Arabidopsis

(a) The column "MS intensity (Blue)" refers to the normalized intensity of the phosphopeptide bearing the indicated phosphosite, "Blue" indicates that the samples were irradiated with blue light (30 μmol m^{-2} s^{-1}) for 30 min prior to sample collection and analyses. The column "Blue/Dark" indicates the ratio of the relative abundance of each phosphopeptide in blue light- and dark-treated samples. The statistical significance of the blue-light responsiveness (Blue/Dark) is indicated by P values.

(b) The precursors selected for Parallel Reaction Monitoring (PRM) are shown, in which phosphorylation is referred to P and oxidation is referred to O in the "Modification" column, with the extra mass brought by the modification included in the parenthesis. The column z indicates the charge states of each precursor**.**

Supplementary Table 2

Primer Name	Primer Sequence (5’-3‘)	Assay
PPK1-RT-F	ATGCCGGAGCTTCGCC	RT-PCR
PPK1-RT-R	TCAAGATACAGTTCGGCCATAG	
PPK2-RT-F	GTGGTGTCGGTAGGGGTCGA	
PPK2-RT-R	AGTGCTTGGAAAGGCGGTG	
PPK3-RT-F	GCCTTGACGAGCAGCCTAA	
PPK3-RT-R	GAGAGTCTCTTGGGTTTCATCGG	
PPK4-RT-F	ATGCCTGAGCTGCGTAGC	
PPK4-RT-R	TCATGACACAGTTCGACCATAAC	
Flag-PPK1-F	GAATTCTGCAGGATCCATGCCGGAGCTTCGCC	Human cell expression vectors
Flag-PPK1-R	TTTAATCGATGTCGACTCAAGATACAGTTCGGCCATAG	
Flag-PPK2-F	GAATTCTGCAGGATCCATGCCAGAGTTAAGAAGTGGAGC	
Flag-PPK2-R	TTTAATCGATGTCGACTCAGCAAACTGTCCTCCCAA	
Flag-PPK3-F	GAATTCTGCAGGATCCATGCCAGAGTTAAGAAGTGGAGC	
Flag-PPK3-R	TTTAATCGATGTCGACTCAGCAAACTGTCCGACCATAG	
Flag-PPK4-F	GAATTCTGCAGGATCCATGCCTGAGCTGCGTAGC	
Flag-PPK4-R	TTTAATCGATGTCGACTCATGACACAGTTCGACCATAAC	
Flag-CK1.3-F	GAATTCTGCAGGATCCATGATAGATACGCTTTTCTTCTTGT	Human cell expression vectors
Flag-CK1.3-R	TTTAATCGATGTCGACTCATTGACTTCTCATTCTGCTG	
Flag-CK1.4-F	GAATTCTGCAGGATCCATGGAACGTATCATCGGCG	
Flag-CK1.4-R	TTTAATCGATGTCGACTCATTTCCCCGGTGACATG	

Flag-MAPK12-F	GAATTCTGCAGGATCCATGTCTGGAGAATCAAGCTCTG	
Flag- MAPK12-R	TTTAATCGATGTCGACTCAGTGGTCAGGATTGAATTTG	
Flag-GFP-PPK1-F	CTCCAGCTCCAGCTGGTACCATGCCGGAGCTTCGCC	
Flag-GFP-PPK1-R	TAAGCGTGCTCAGCGGTACCAGATACAGTTCGGCCATAGCTT	
Flag-GFP-PPK2-F	CTCCAGCTCCAGCTGGTACCATGCCAGAGTTAAGAAGTGGAGC	
Flag-GFP-PPK2-R	TAAGCGTGCTCAGCGGTACCGCAAACTGTCCTCCCAAAGC	
Flag-GFP-PPK3-F	CTCCAGCTCCAGCTGGTACCATGCCAGAGTTAAGAAGTGGAGC	
Flag-GFP-PPK3-R	TAAGCGTGCTCAGCGGTACCTCAGCAAACTGTCCGACCATAG	
Flag-GFP-PPK4-F	CTCCAGCTCCAGCTGGTACCATGCCTGAGCTGCGTAGC	
Flag-GFP-PPK4-R	TAAGCGTGCTCAGCGGTACCTCATGACACAGTTCGACCATAAC	
GFP-CRY2-F	CTCCAGCTCCAGCTGGTACCATGAAGATGGACAAAAAGACTATAG	
GFP-CRY2-R	TAAGCGTGCTCAGCGGTACCTCATTTGCAACCATTTTTTC	
Myc-CRY2-F	GGAGGACCTGGGATCCATGAAGATGGACAAAAAGACTATAG	
Myc-CRY2-F	TAGCAGGCCTGGATCCTCATTTGCAACCATTTTTTC	
BD-CRY2-F	TGTATCGCCGGAATTCATGAAGATGGACAAAAAGACTATAG	
BD-CRY2-F	TTGGCTGCAGGTCGACTCATTTGCAACCATTTTTTC	Yeast two-hybrid
AD-PPK1-F	GGAGGCCAGTGAATTCATGCCGGAGCTTCGCC	
AD-PPK1-R	TCATCTGCAGCTCGAGTCAAGATACAGTTCGGCCATAG	
AD-PPK2-F	GGAGGCCAGTGAATTCATGCCAGAGTTAAGAAGTGGAGC	
AD-PPK2-R	TCATCTGCAGCTCGAGTCAGCAAACTGTCCTCCCAA	
AD-PPK3-F	GGAGGCCAGTGAATTCATGCCAGAGTTAAGAAGTGGAGC	Yeast two-hybrid
AD-PPK3-R	TCATCTGCAGCTCGAGTCAGCAAACTGTCCGACCATAG	

AD-PPK4-F	GGAGGCCAGTGAATTCATGCCTGAGCTGCGTAGC	
AD-PPK4-R	TCATCTGCAGCTCGAGTCATGACACAGTTCGACCATAAC	
AD-PPK1C-F	GGAGGCCAGTGAATTCCAAGTTGGCCAGAAGCGA	
AD-PPK1C-R	TCATCTGCAGCTCGAGTCAAGATACAGTTCGGCCATAG	
AD-PPK2C-F	GGAGGCCAGTGAATTCGAACAATGTGCACTATCTAGACCA	
AD-PPK2C-R	TCATCTGCAGCTCGAGTCAGCAAACTGTCCTCCCA	
AD-PPK3C-F	GGAGGCCAGTGAATTCGAGCCATGTGCTATATCTAGACCAA	
AD-PPK3C-R	TCATCTGCAGCTCGAGTCAGCAAACTGTCCGACCATAG	
AD-PPK4C-F	GGAGGCCAGTGAATTCCAAAAGAGGGGGAGGCTGA	
AD-PPK4C-R	TCATCTGCAGCTCGAGTCATGACACAGTTCGACCATAACA	
FT-Qpcr-F	CAACCCTCACCTCCGAGAATAT	RT-qPCR
FT-Qpcr-F	TTGCCAAAGGTTGTTCCAGTT	
IPP2-Qpcr-F	CTCCCTTGGGACGTATGCTG	
IPP2-Qpcr-R	TTGAACCTTCACGTCTCGCA	

Supplementary Table 2 Primer used in this study.
F, forward primer; R, reverse primer.

Supplementary Figure 1

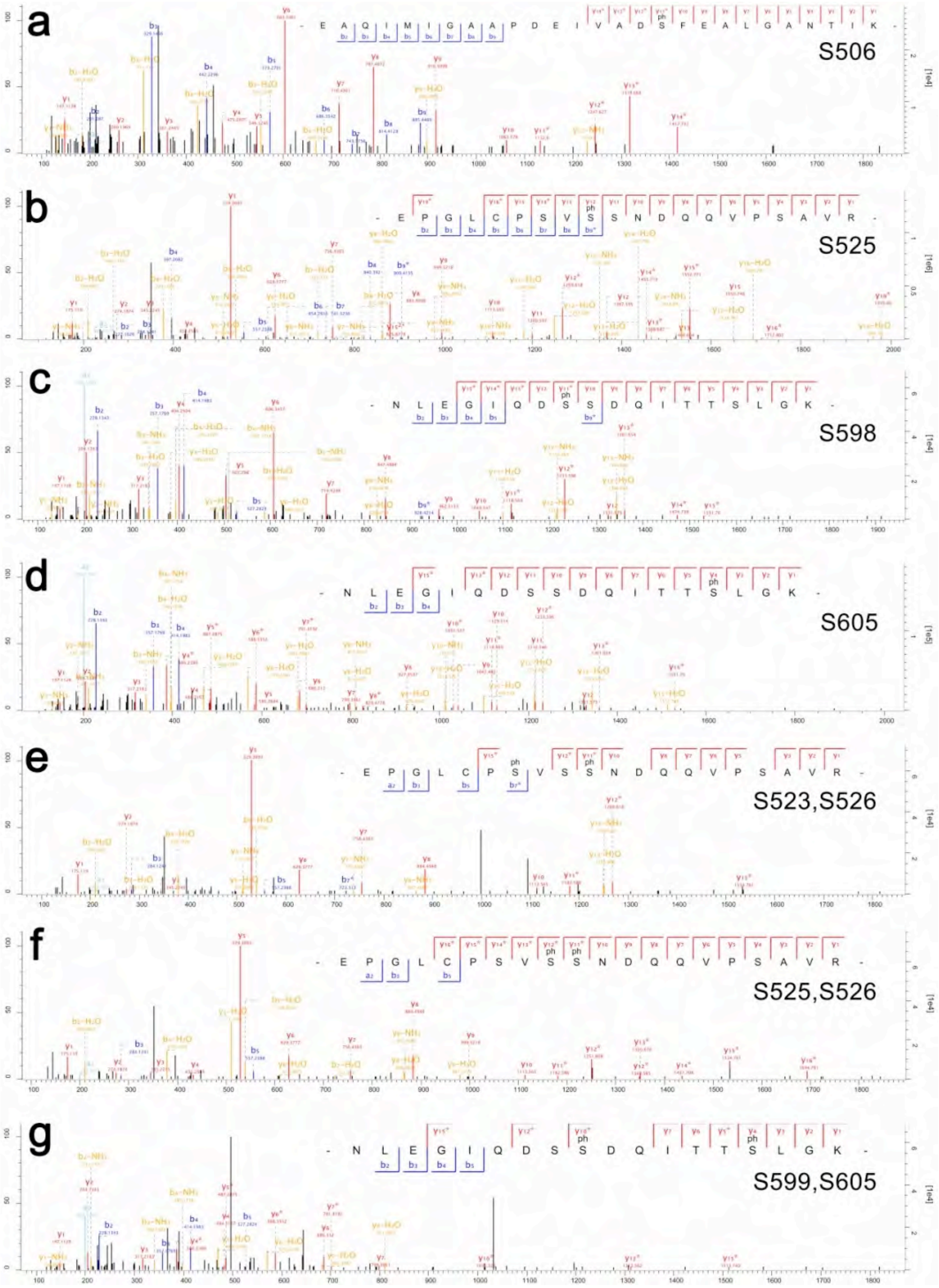

Supplementary Figure 1. Higher-energy Collisional Dissociation (HCD) spectra of the phosphopeptides identified by mass spectrometry. The phosphosites in each phosphopeptide are indicated.

Supplementary Figure 2

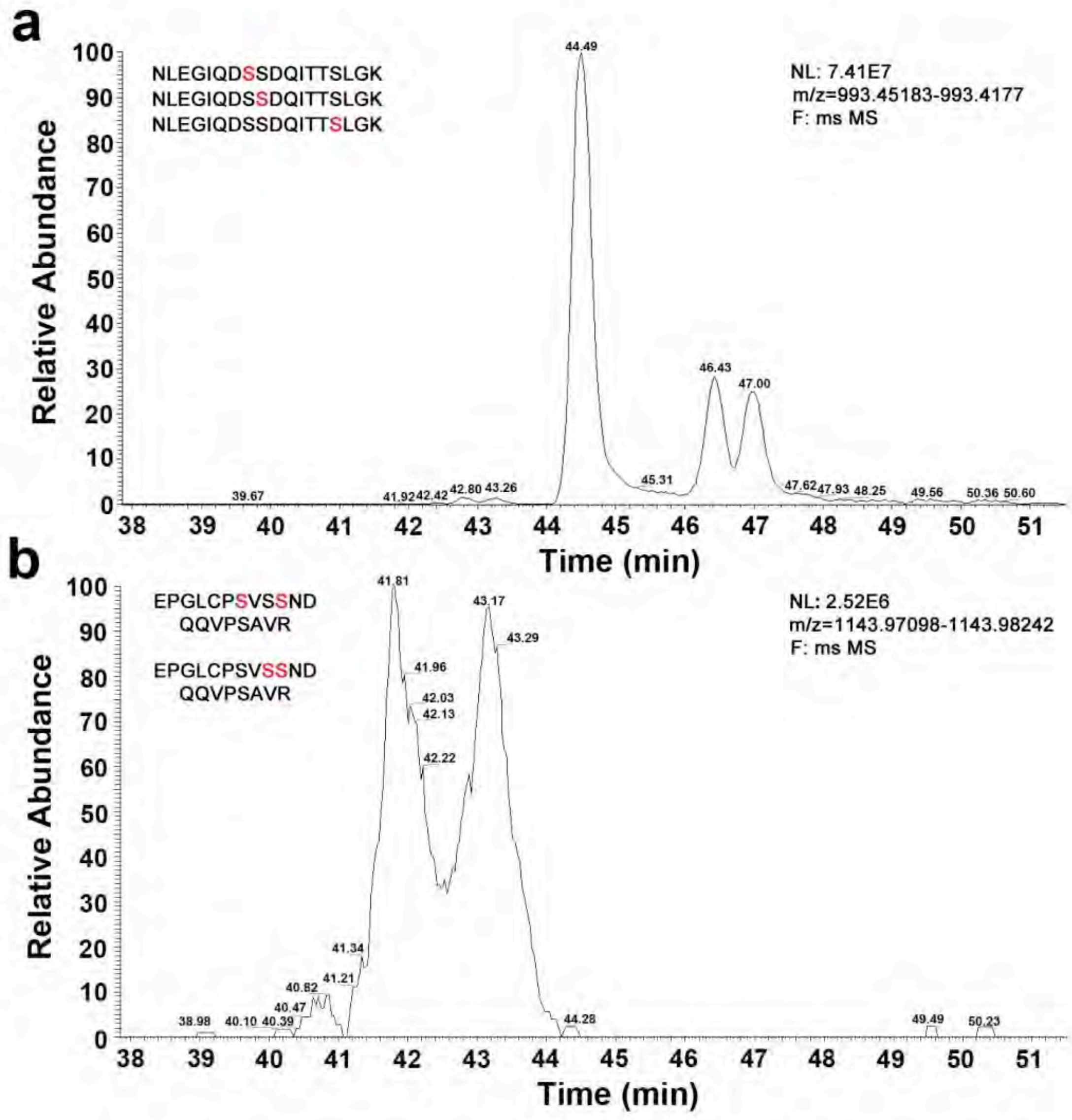

Supplementary Figure 2 Spectrum of phosphopeptides extracted by the X-Calibre software

The different isomers (different MS1 peaks) of the phosphopeptide with same M/Z separated with different retention time in MS1. (**a**) Spectrum of phosphopeptide NLEGIQDSSDQITTSLGK, (**b)** Spectrum of phosphopeptide EPGLCPSVSSNDQQVPSAVR.

Supplementary Figure 3

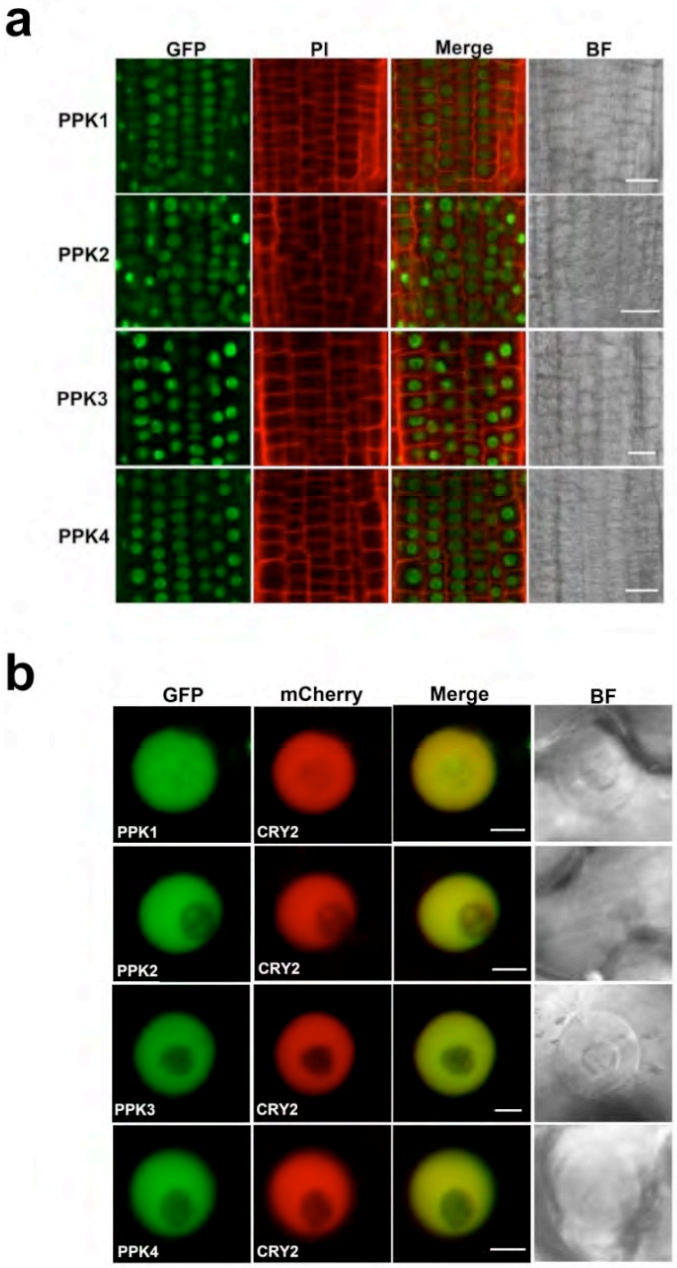

Supplementary Figure 3. PPKs are nuclear proteins co-localize with CRY2 in plants
(a) Subcellular localizations of GFP-PPK1, GFP-PPK2, GFP-PPK3, or GFP-PPK4 fusion proteins in 5-day-old LD-grown transgenic seedlings constitutively expressing the indicated GFP-PPK. The cell walls were visualized by staining with propidium iodide (PI). BF, Bright Field; Scale bars = 20 µm. (**b**) Nuclear co-localizations of GFP-PPKs with CRY2-mCherry transiently expressed in leaves of *Nicotiana benthamiana*. Scale bars = 5 µm.

Supplementary Figure 4

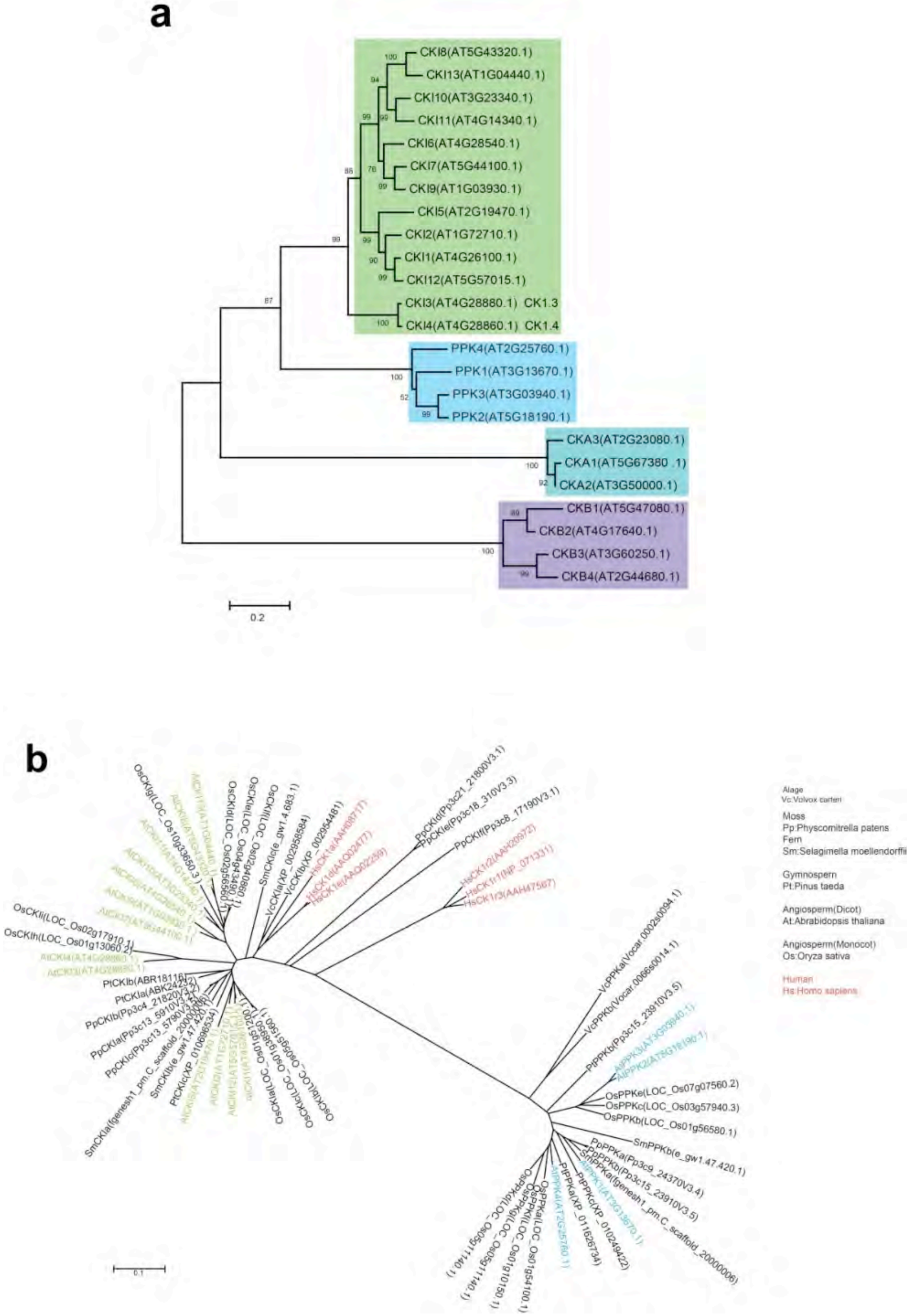

Supplementary Figure 4. Phylogenetic analysis of PPKs and Casein Kinases (CKs)

(a) An un-rooted phylogenetic tree was generated by the neighbor-joining method using the amino acid sequences of PPKs and CKs from Arabidopsis. Different clades are shaded by designated colors. The bar indicates substitution per site.

(b) An un-rooted phylogenetic tree was generated by the neighbor-joining method using the amino acid sequences of PPKs and CK1s from indicated species. Genus/species abbreviations are shown in the figure. The scale bar indicates substitution per site. Arabidopsis PPKs, CK1s and human CK1s are highlighted by indicated colors.

Supplementary Figure 5

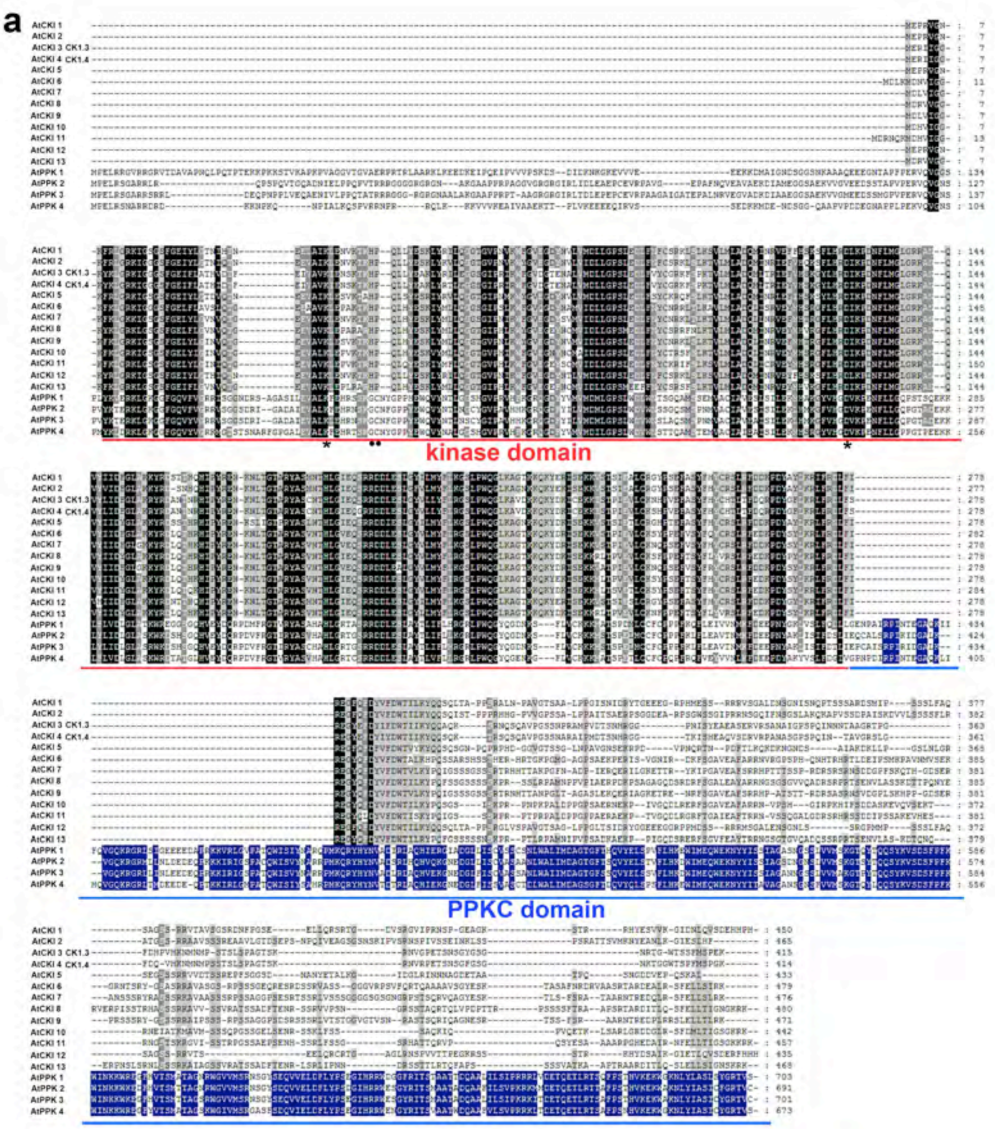

b

Percent Identity (upper triangle); Divergence (lower triangle)

	1	2	3	4	5	6	7	8	9	10	11	12	13	14	15	16	17		
1		92.6	74.8	73.7	85.6	78.9	83.0	75.9	83.3	80.4	80.0	95.9	76.7	38.7	41.7	40.6	41.0	1	CK1.1
2	7.8		75.8	75.1	86.2	78.4	82.9	77.3	84.0	80.7	79.6	90.3	77.0	39.2	41.1	40.0	40.8	2	CK1.2
3	30.7	29.2		97.8	74.4	75.6	76.3	73.0	74.4	73.3	71.1	75.9	73.0	39.5	39.8	39.8	40.2	3	CK1.3
4	32.4	30.3	2.3		73.0	75.6	74.4	72.6	72.6	73.0	71.5	75.6	71.9	39.8	39.5	39.5	39.8	4	CK1.4
5	16.1	15.2	31.3	33.5		75.9	78.9	76.3	80.4	77.8	78.1	84.1	75.9	37.6	40.2	39.8	41.4	5	CK1.5
6	24.8	25.5	29.6	29.6	29.1		89.3	86.3	88.5	86.3	84.8	78.9	83.7	38.7	39.5	38.7	41.0	6	CK1.6
7	19.4	19.5	28.5	31.3	24.8	11.6		84.1	94.8	87.4	84.4	82.2	83.7	38.0	38.7	38.0	40.2	7	CK1.7
8	29.1	27.1	33.5	34.1	28.5	15.2	18.0		85.6	88.1	87.0	76.7	94.8	38.7	39.5	38.7	40.6	8	CK1.8
9	18.9	18.0	31.3	34.1	22.8	12.5	5.4	16.1		88.1	85.2	82.2	84.1	38.7	39.5	38.0	41.7	9	CK1.9
10	22.8	22.4	33.0	33.5	26.4	15.2	13.8	12.9	12.9		91.1	80.7	87.8	39.1	39.8	39.5	42.5	10	CK1.10
11	23.3	23.9	36.5	35.9	25.9	17.0	17.5	14.3	16.6	9.5		80.0	86.3	39.1	39.8	39.1	42.5	11	CK1.11
12	4.2	10.4	29.1	29.6	18.0	24.8	20.3	28.0	20.3	22.3	23.3		76.7	39.1	41.0	39.8	40.6	12	CK1.12
13	28.0	27.6	33.5	35.3	29.1	18.4	18.4	5.4	18.0	13.4	15.2	28.0		38.0	40.2	39.5	40.2	13	CK1.13
14	116.4	114.4	113.5	112.0	121.0	116.4	119.5	116.4	116.4	114.9	114.9	114.9	119.5		77.6	75.3	78.0	14	PPK1
15	105.2	107.3	112.0	113.5	110.6	113.5	116.4	113.5	113.5	112.0	112.0	107.9	110.6	26.6		94.9	72.9	15	PPK2
16	109.2	111.5	112.0	113.5	112.0	116.4	119.5	116.4	119.5	113.5	114.9	112.0	113.5	30.1	5.3		72.2	16	PPK3
17	107.9	108.7	110.6	112.0	106.5	107.9	110.6	109.2	105.2	102.5	102.5	109.2	110.6	26.1	33.7	34.7		17	PPK4
	1	2	3	4	5	6	7	8	9	10	11	12	13	14	15	16	17		

Supplementary Figure 5. Amino acid sequence alignment of PPKs and Casein Kinase 1 (CK1)
(a) The full-length amino acid sequences of PPKs and CK1s are aligned using ClustalX and manually adjusted. The numbers indicate positions of the amino acid. The red and blue underlines indicate the sequences encoding the kinase and PPK C-terminal (PPKC) domains, respectively. In the kinase domain, two residues (K175 and D267 of PPK1) that are conserved among all PPKs and CK1s are labeled by asterisks, and two residues (G183 and C184 of PPK1) that are conserved only in PPKs are indicated by black dots underneath. (**b**) The percent identity and divergence between the kinase domain of PPKs and CKIs. The amino acid sequences of the kinase domain of PPKs and CKIs are aligned using MegAlign.

Supplementary Figure 6

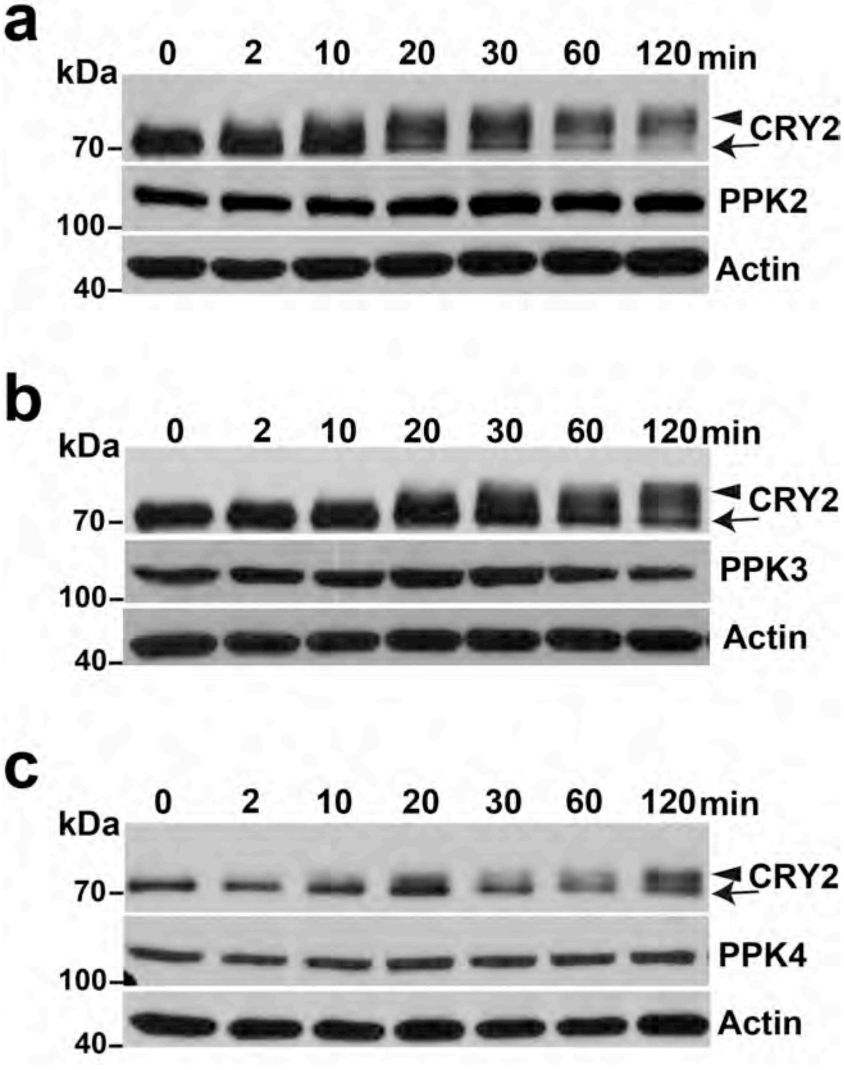

Supplementary Figure 6 Immunoblot showing the time course of light-dependent phosphorylation of CRY2 by PPKs

HEK293T cells co-expressing *CMV::Myc-CRY2* and *CMV::Flag-PPK2* (a), or *Flag-PPK3* (b) or *Flag-PPK4* (c) were exposed to blue light (30 µmol m^{-2} s^{-1}) for the indicated time (0 to 120 min). CRY2 and PPKs were detected by immunoblots probed with anti-Myc or anti-Flag antibodies, respectively. In this and other figures alike, arrows or arrowheads indicate unphosphorylated or phosphorylated CRY2, respectively, unless otherwise indicated. Actin or a non-specific band (NS) recognized by anti-Flag antibody was used as the loading control.

Supplementary Figure 7

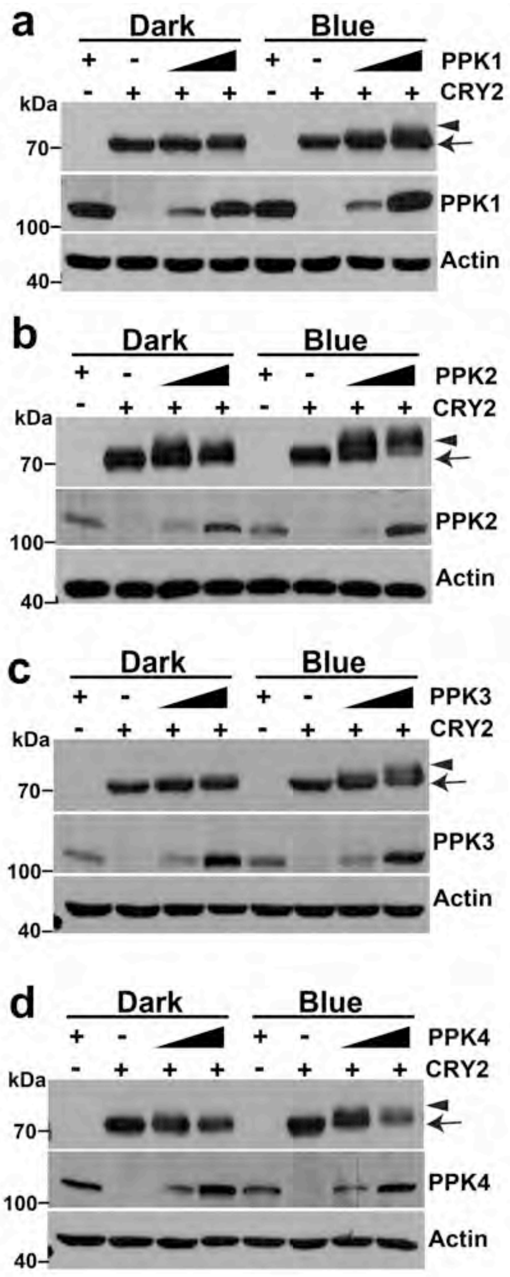

Supplementary Figure 7 The blue light-dependent phosphorylation of CRY2 is dependent on the dosage of PPKs

a-d, Immunoblot assays showing the dosage effect of PPK1(a), PPK2 (b), PPK3 (c) and PPK4 (d) on blue light-dependent phosphorylation of CRY2 in HEK293T cells. Cells transfected to express *CMV::Myc-CRY2*, *CMV::Flag-GFP-PPKs* or both were kept in the dark or exposed to the blue light (30 μmol m^{-2} s^{-1}) for 30 min. The black triangle on the top indicates the increasing amount of plasmids encoding indicated PPKs used in transfection. CRY2 and PPKs were detected by immunoblots probed with anti-Myc or anti-Flag antibodies, respectively. The arrowhead and arrow indicate the phosphorylated CRY2 (upshift bands) and unphosphorylated CRY2, respectively. Actin was used as loading control.

Supplementary Figure 8

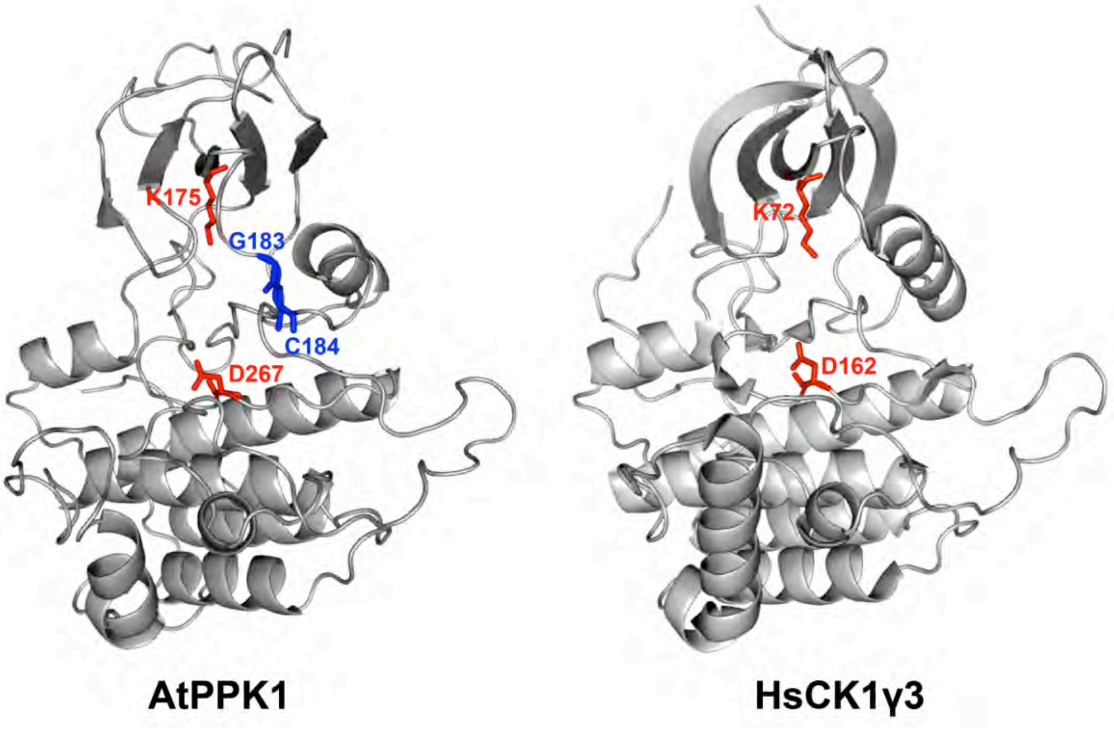

Supplementary Figure 8. A comparison of 3D structures of the kinase domain of human CK1γ3 [Protein Data Bank (PDB) ID code 2CHL] (Right) and the modeled structure of the kinase domain of Arabidopsis PPK1 (Left). Residues K175 of Arabidopsis PPK1 and K72 of human CK1γ3 are equivalent to the referenced K38 of CK1ε in human; D267 of Arabidopsis PPK1 and D162 of human CK1γ3 are equivalent to the referenced D131 of CK1 in *Xenopus laevis*. G183 and C184 of Arabidopsis PPK1 are the residues only conserved in PPKs but not in CK1s.

Supplementary Figure 9

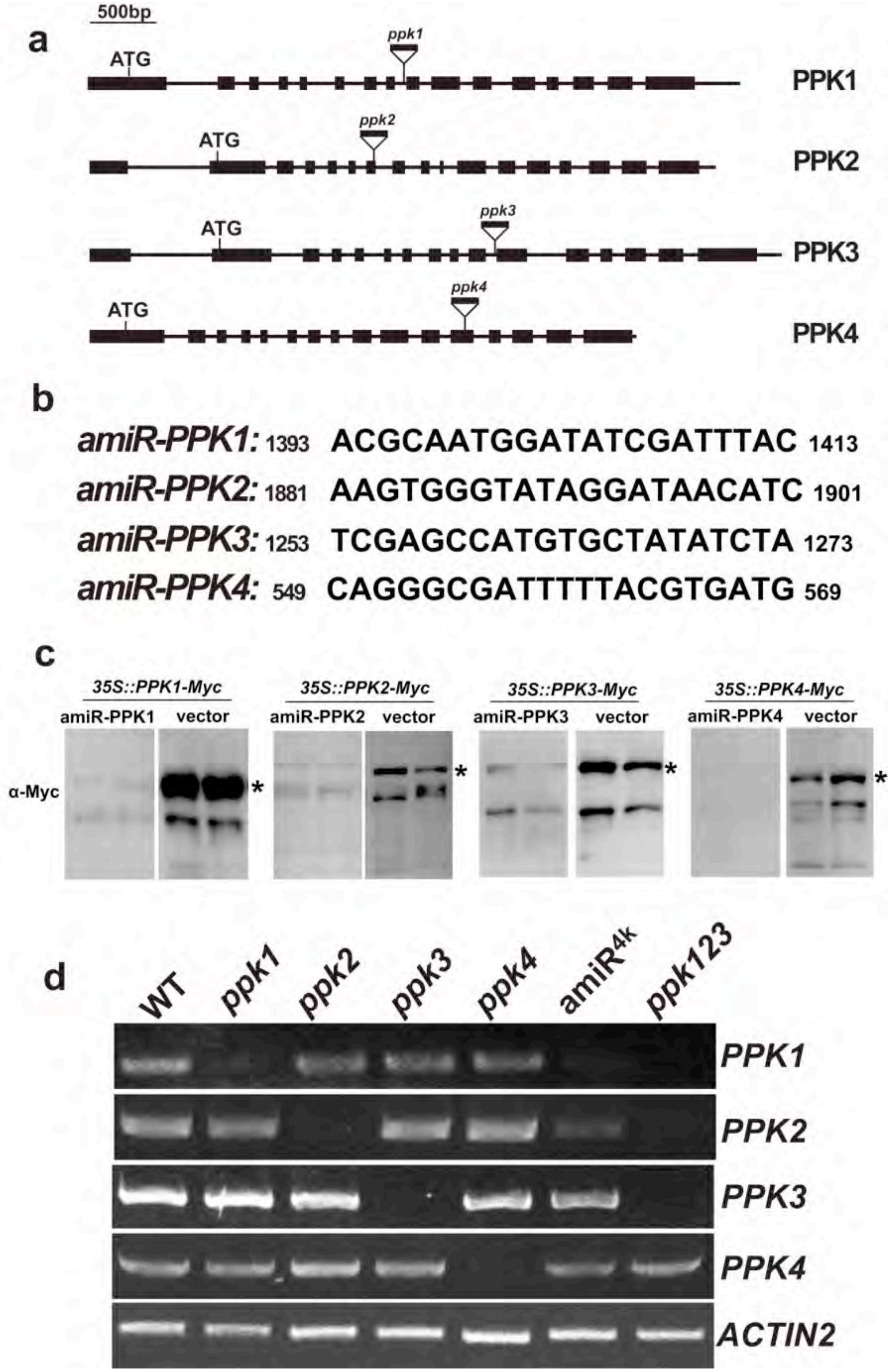

Supplementary Figure 9. Genotyping of *ppk123* and *ppk124* triple mutants and analysis of the amiRNA^{4K} transgenic line

(a) Schematic diagram of structures of the *PPK* genes and the corresponding mutants. Black boxes and lines indicate exon and intron (or 3'UTR) regions, respectively. T-DNA insertions in the indicated *ppk* single mutants are shown as triangles. **(b)** Target sequence of the individual *PPK* genes targeted by artificial microRNAs. Numbers indicate positions of the start and the end of the target DNA fragment of the indicated *PPK* gene. **(c)** Immunoblot showing the silencing effect of indicated artificial microRNAs on the protein expression of PPKs. The *Agrobacteria* strain harboring indicated Ti plasmid encoding individual Myc-PPKs were transfected alone to leaves of *Nicotiana benthamiana* (vector) or co-transfected with the *Agrobacterial* strains harboring the Ti plasmids expressing indicated artificial microRNAs (amiR-PPK). After transfection, proteins of respective samples were analyzed by immunoblot probed with anti-Myc antibodies. **(d)** RT-PCR (Reverse Transcription-PCR) analyses of mRNA levels of the *PPK* genes in wild type (WT), *ppk* monogenic mutants (including *ppk1*, *ppk2*, *ppk3,* and *ppk4*), the *ppk123* triple mutant, and an amiRNA^{4k} line (#1). The mRNA expression of *Actin2* was used as the loading control.

Supplementary Figure 10

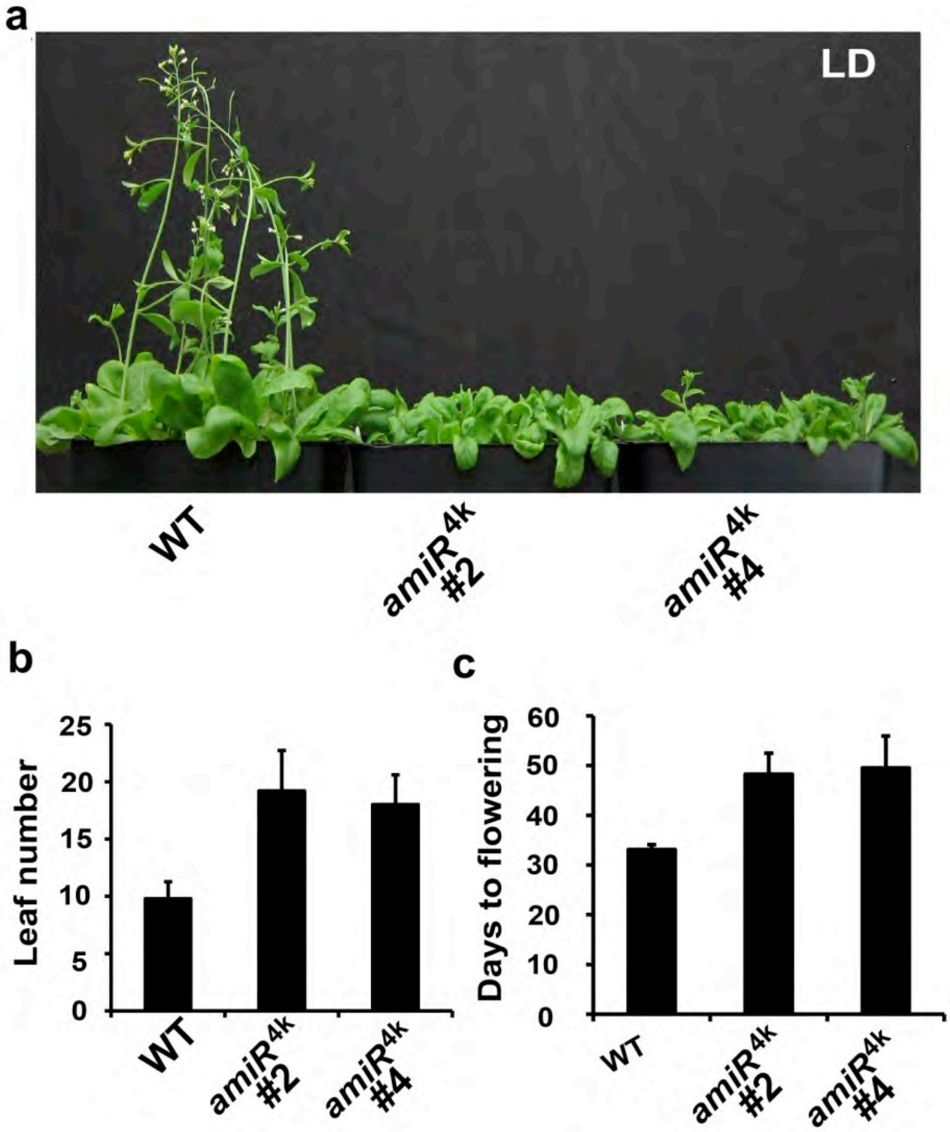

Supplementary Figure 10. Independent transgenic lines expressing the artificial microRNA ($amiRNA^{4K}$) showed delayed flowering in long-day photoperiods (LD)

(a) Images of wild-type (WT) plants and two PPKs artificial microRNA transgenic lines ($amiRNA^{4K}$ #2 and #4) grown in long day photoperiods (16 hr light, 8 hr dark) for 27 days. (**b-c)** Flowering time of the indicated genotypes measured as “leaf number” (b) or “Days to flowering” (c) are shown with standard deviations (n>20).

Supplementary Figure 11

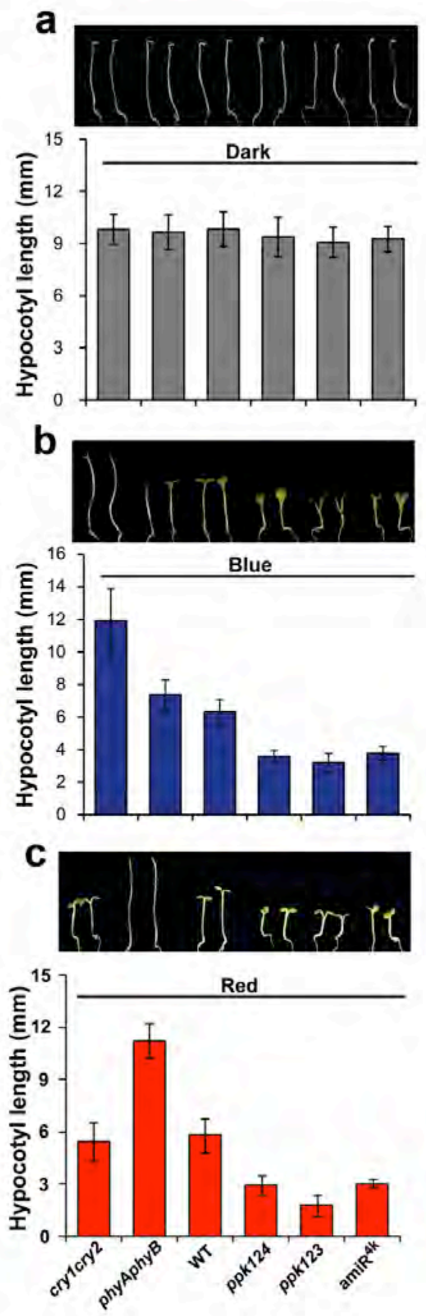

Supplementary Figure 11. Photomorphogenic analyses of *ppk* mutants and the transgenic plants expressing artificial microRNA targeting all four PPKs (amiRNA^{4K}) Representative images of seedlings (top) and hypocotyl lengths (bottom) of the *ppk* triple mutants (*ppk123* and *ppk124*) and transgenic plants expressing the artificial microRNA amiRNA^{4K}. Wild-type (WT), *cry1cry2* and *phyAphyB* mutants were included as the controls. Plants were grown in **(a)** dark, **(b)** continuous blue light (10µmol $m^{-2}s^{-1}$), or **(c)** red light (20µmol $m^{-2}s^{-1}$). Standard deviations are shown (SD, n>20).

Supplementary Figure 12

Fig.1c

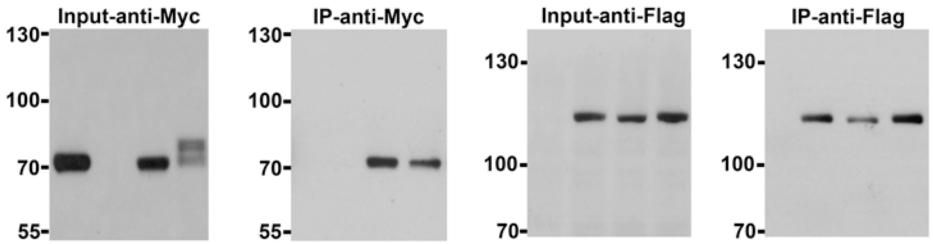

Fig.1d

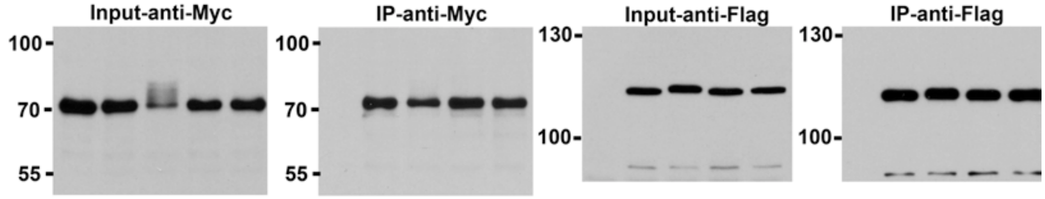

Fig.1e

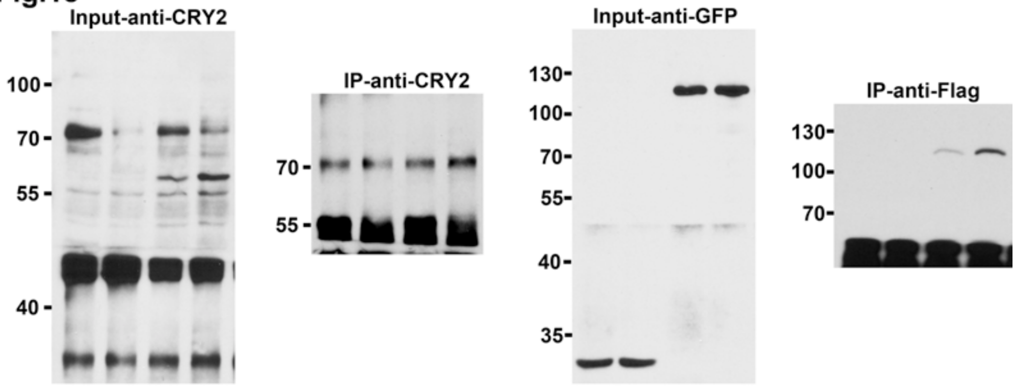

Fig.3a

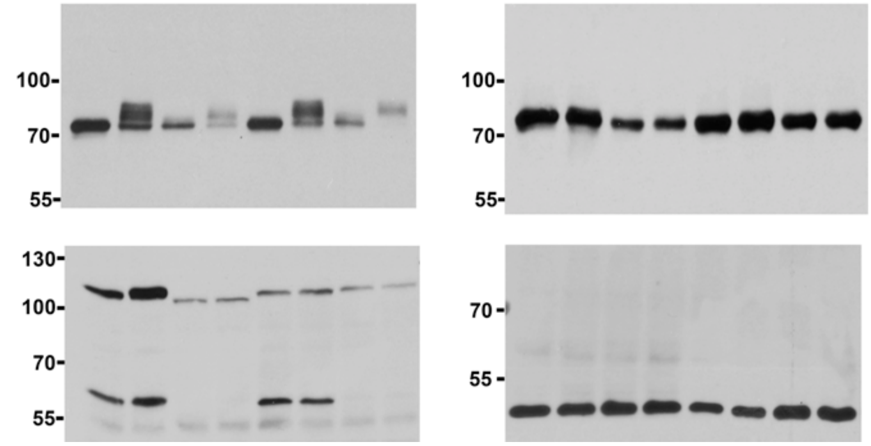

Fig.3b

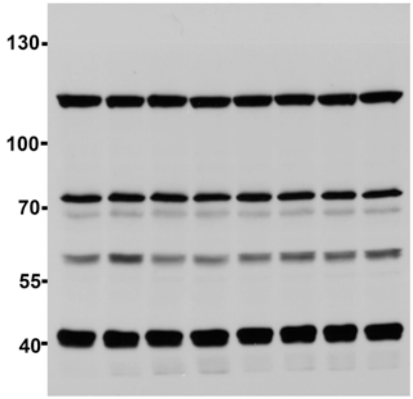

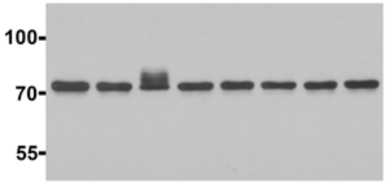

Fig.4a

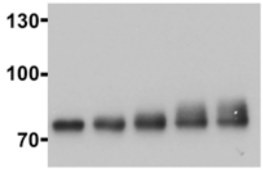

Fig.3d-e

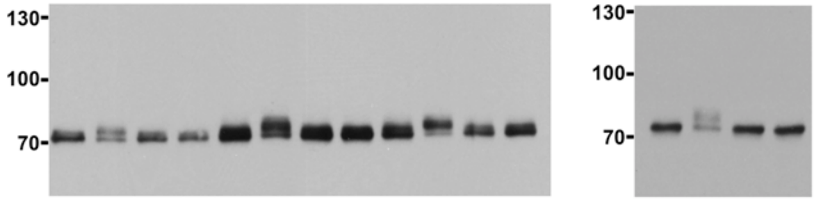

Fig.4b& Fig.S6a

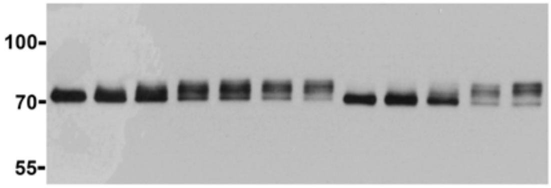

Fig.4c

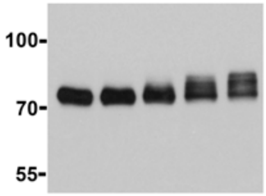

Fig.4d& Fig.S6c

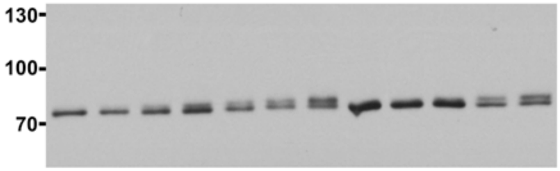

Fig.5a& Fig.S6b

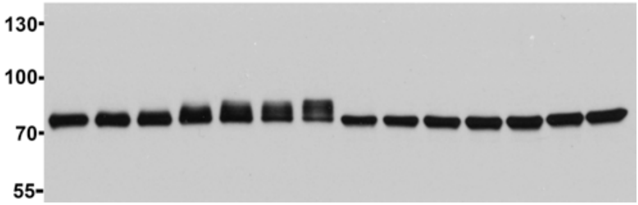

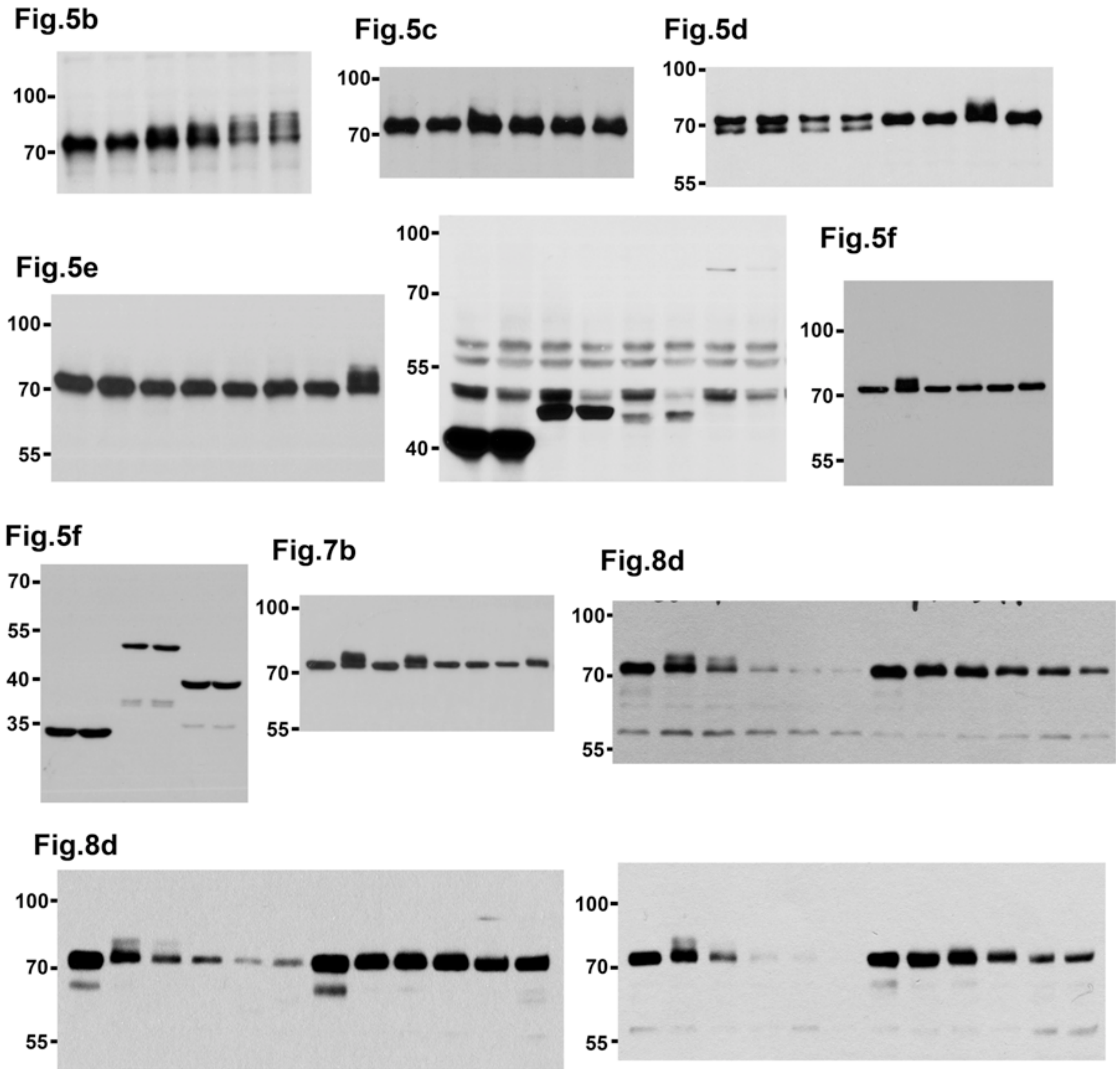

Supplementary Figure 12. The original full images of immunoblots used to prepare figures.
